# The Effect of Tobacco Control Measures during a Period of Rising Cardiovascular Disease Risk in India: A Mathematical Model of Myocardial Infarction and Stroke

**DOI:** 10.1371/journal.pmed.1001480

**Published:** 2013-07-09

**Authors:** Sanjay Basu, Stanton Glantz, Asaf Bitton, Christopher Millett

**Affiliations:** 1 Prevention Research Center, Department of Medicine, Stanford University, Stanford, California, United States of America; 2 Centers for Health Policy, Primary Care and Outcomes Research, Stanford University, Stanford, California, United States of America; 3 Center on Poverty and Inequality, Stanford University, Stanford, California, United States of America; 4 Department of Public Health and Policy, London School of Hygiene and Tropical Medicine, London, United Kingdom; 5 Department of Medicine, University of California San Francisco, San Francisco, California, United States of America; 6 Division of General Medicine, Brigham and Women's Hospital, Boston, Massachusetts, United States of America; 7 Department of Health Care Policy, Harvard Medical School, Boston, Massachusetts, United States of America; 8 School of Public Health, Imperial College London, London, United Kingdom; 9 South Asia Network for Chronic Disease, Public Health Foundation of India, New Delhi, India; University of Southern California, United States of America

## Abstract

**Background:**

We simulated tobacco control and pharmacological strategies for preventing cardiovascular deaths in India, the country that is expected to experience more cardiovascular deaths than any other over the next decade.

**Methods and Findings:**

A microsimulation model was developed to quantify the differential effects of various tobacco control measures and pharmacological therapies on myocardial infarction and stroke deaths stratified by age, gender, and urban/rural status for 2013 to 2022. The model incorporated population-representative data from India on multiple risk factors that affect myocardial infarction and stroke mortality, including hypertension, hyperlipidemia, diabetes, coronary heart disease, and cerebrovascular disease. We also included data from India on cigarette smoking, bidi smoking, chewing tobacco, and secondhand smoke. According to the model's results, smoke-free legislation and tobacco taxation would likely be the most effective strategy among a menu of tobacco control strategies (including, as well, brief cessation advice by health care providers, mass media campaigns, and an advertising ban) for reducing myocardial infarction and stroke deaths over the next decade, while cessation advice would be expected to be the least effective strategy at the population level. In combination, these tobacco control interventions could avert 25% of myocardial infarctions and strokes (95% CI: 17%–34%) if the effects of the interventions are additive. These effects are substantially larger than would be achieved through aspirin, antihypertensive, and statin therapy under most scenarios, because of limited treatment access and adherence; nevertheless, the impacts of tobacco control policies and pharmacological interventions appear to be markedly synergistic, averting up to one-third of deaths from myocardial infarction and stroke among 20- to 79-y-olds over the next 10 y. Pharmacological therapies could also be considerably more potent with further health system improvements.

**Conclusions:**

Smoke-free laws and substantially increased tobacco taxation appear to be markedly potent population measures to avert future cardiovascular deaths in India. Despite the rise in co-morbid cardiovascular disease risk factors like hyperlipidemia and hypertension in low- and middle-income countries, tobacco control is likely to remain a highly effective strategy to reduce cardiovascular deaths.

*Please see later in the article for the Editors' Summary*

## Abstract 2

In this paper from Basu and colleagues, a simulation of tobacco control and pharmacological interventions to prevent cardiovascular disease mortality in India predicted that Smokefree laws and increased tobacco taxation are likely to be the most effective measures to avert future cardiovascular deaths in India.

*Please see later in the article for the Editors' Summary*

## Editors' Summary

**Background:**

Cardiovascular diseases (CVDs) are conditions that affect the heart and/or the circulation. In coronary heart disease, for example, narrowing of the heart's blood vessels by fatty deposits slows the blood supply to the heart and may eventually cause a heart attack (myocardial infarction). Stroke, by contrast, is a CVD in which the blood supply to the brain is interrupted. CVD has been a major cause of illness and death in high-income countries for many years, but the burden of CVD is now rapidly rising in low- and middle-income countries. Indeed, worldwide, three-quarters of all deaths from heart disease and stroke occur in low- and middle-income countries. Smoking, high blood pressure (hypertension), high blood cholesterol (hyperlipidemia), diabetes, obesity, and physical inactivity all increase an individual's risk of developing CVD. Prevention strategies and treatments for CVD include lifestyle changes (for example, smoking cessation) and taking drugs that lower blood pressure (antihypertensive drugs) or blood cholesterol levels (statins) or thin the blood (aspirin).

**Why Was This Study Done?:**

Because tobacco use is a key risk factor for CVD and for several other noncommunicable diseases, the World Health Organization has developed an international instrument for tobacco control called the Framework Convention on Tobacco Control (FCTC). Parties to the FCTC (currently 176 countries) agree to implement a set of core tobacco control provisions including legislation to ban tobacco advertising and to increase tobacco taxes. But will tobacco control measures reduce the burden of CVD effectively in low- and middle-income countries as other risk factors for CVD are becoming more common? In this mathematical modeling study, the researchers investigated this question by simulating the effects of tobacco control measures and pharmacological strategies for preventing CVD on CVD deaths in India. Notably, many of the core FCTC provisions remain poorly implemented or unenforced in India even though it became a party to the convention in 2005. Moreover, experts predict that, over the next decade, this middle-income country will contribute more than any other nation to the global increase in CVD deaths.

**What Did the Researchers Do and Find?:**

The researchers developed a microsimulation model (a computer model that operates at the level of individuals) to quantify the likely effects of various tobacco control measures and pharmacological therapies on deaths from myocardial infarction and stroke in India between 2013 and 2022. They incorporated population-representative data from India on risk factors that affect myocardial infarction and stroke mortality and on tobacco use and exposure to secondhand smoke into their model. They then simulated the effects of five tobacco control measures—smoke-free legislation, tobacco taxation, provision of brief cessation advice by health care providers, mass media campaigns, and advertising bans—and increased access to aspirin, antihypertensive drugs, and statins on deaths from myocardial infarction and stroke. Smoke-free legislation and tobacco taxation are likely to be the most effective strategies for reducing myocardial infarction and stroke deaths over the next decade, according to the model, and the effects of these strategies are likely to be substantially larger than those achieved by drug therapies under current health system conditions. If the effects of smoke-free legislation and tobacco taxation are additive, the model predicts that these two measures alone could avert about 9 million deaths, that is, a quarter of the expected deaths from myocardial infarction and stroke in India over the next 10 years, and that a combination of tobacco control policies and pharmacological interventions could avert up to a third of these deaths.

**What Do These Findings Mean?:**

These findings suggest that the implementation of smoke-free laws and the introduction of increased tobacco taxes in India would yield substantial and rapid health benefits by averting future CVD deaths. The accuracy of these findings is likely to be affected by the many assumptions included in the mathematical model and by the quality of the data fed into it. Importantly, however, these finding suggest that, despite the rise in other CVD risk factors such as hypertension and hyperlipidemia, tobacco control is likely to be a highly effective strategy for the reduction of CVD deaths over the next decade in India and probably in other low- and middle-income countries. Policymakers in these countries should, therefore, work towards fuller and faster implementation of the core FCTC provisions to boost their efforts to reduce deaths from CVD.

**Additional Information:**

Please access these websites via the online version of this summary at http://dx.doi.org/10.1371/journal.pmed.1001480.

- The American Heart Association provides information on all aspects of cardiovascular disease; its website includes personal stories about heart attacks and stroke
- The US Centers for Disease Control and Prevention has information on heart disease and on stroke (in English and Spanish
- The UK National Health Service Choices website provides information about cardiovascular disease and stroke
- MedlinePlus provides links to other sources of information on heart diseases, vascular diseases, and stroke (in English and Spanish)
- The World Health Organization provides information (in several languages) about the dangers of tobacco, about the Framework Convention on Tobacco Control, and about noncommunicable diseases; its Global Noncommunicable Disease Network (NCDnet) aims to help low- and middle- income countries reduce illness and death caused by CVD and other noncommunicable diseases
- SmokeFree, a website provided by the UK National Health Service, offers advice on quitting smoking and includes personal stories from people who have stopped smoking
- Smokefree.gov, supported by the US National Cancer Institute and other US agencies, offers online tools and resources to help people quit smoking

## Introduction

The burden of cardiovascular disease (CVD) is rapidly rising in low- and middle-income countries (LMICs) [1]. LMICs now account for over three-quarters of all global deaths from heart disease and stroke [2], with India expected to contribute more than any other nation to the growth in these deaths over the decade from 2010 to 2020. More than one in three CVD deaths in India are projected to occur among young and working-age people, leading to substantial economic and societal costs [3]. The United Nations 2011 High Level Meeting on Prevention and Control of Non-Communicable Diseases identified tobacco use as a key risk factor for noncommunicable diseases [4],[5]. Tobacco-related deaths are projected to double in LMICs between 2000 and 2030 [1]. Smoking has been estimated to be responsible for about one in 20 deaths among Indian women and one in five deaths among Indian men aged 30 to 69 y [6], many due to CVD. Due in part to tobacco use, CVD deaths are expected to increase approximately 12% over the next decade [5].

Recognizing the burden of disease attributable to tobacco and the cost-effectiveness of tobacco control measures [4], India and 175 other countries ratified the World Health Organization (WHO) Framework Convention on Tobacco Control (FCTC) [7]. The FCTC commits nations to enact tobacco control legislation fulfilling minimum standards for the production, sale, distribution, advertisement, and taxation of tobacco, as well as legislation protecting populations from secondhand smoke [7]. Major FCTC provisions include smoke-free laws, brief cessation advice by health care providers, mass media campaigns, a tobacco advertising ban, and increased tobacco taxes.

Several questions have been recently raised, however, about the effectiveness of tobacco control measures in LMICs. It remains unclear how tobacco control will affect future CVD in the setting of emerging co-morbid factors such as hypertension, obesity, and diabetes (the concern being that tobacco control efforts could have diminishing returns in the context of multiple co-morbid risks) [8]. Previous mathematical models of tobacco control have not accounted for these co-morbid conditions, which pose competing risks for CVD death [9],[10]. Only recently have population-representative data become available from developing countries like India, from which we can understand the prevalence rates of numerous CVD risk factors including smoking, hypertension, hyperlipidemia, and diabetes, among both urban and rural populations and among both men and women in multiple age groups [11].

While India passed national tobacco control legislation in 2003 (the Cigarettes and Other Tobacco Products Act) before it ratified the FCTC, many of the core FCTC provisions remain poorly implemented or unenforced. For example, smoke-free legislation has not been consistently implemented; one in three adults reported being exposed to smoking at work in 2009 and 2010, varying from 15.4% in Chandigarh to 67.9% in Jammu and Kashmir [12]. Tobacco cessation programs have received limited government financial support, and cessation advice by health care professionals is provided infrequently [12],[13]. Tobacco taxation remains very low, at around 38% of cigarette and 9% of bidi prices [14], far below the minimum of 70% the WHO recommends [15].

This paper estimates the impact of implementing key FCTC policies on CVD mortality in India using a mathematical model to calculate the number of deaths from myocardial infarction or stroke among Indian adults over the decade from 2013 through 2022. Moving beyond prior models of tobacco control, our model incorporates the effects of co-morbid cardiovascular risk factors to capture complex demographic patterns of concurrent tobacco use and other key cardiovascular risk factors. Further, we examine tobacco use in its myriad of forms common in India, not just cigarette smoking. We also employ new population-representative, age-stratified data from India on both urban and rural prevalence rates in risk factors such as systolic blood pressure, total cholesterol, and diabetes among both men and women. This approach allows us to estimate the added value of tobacco control in an era where other major risk factors are also expected to contribute significantly to CVD mortality.

## Methods

### Modeling Approach and Underlying Assumptions

We constructed a discrete-time microsimulation model of myocardial infarction and stroke mortality among Indian residents aged 20 to 79 y over the decade from 1 January 2013 through 31 December 2022. The model is stratified into 24 cohorts defined by 10-y age groups (20–29, 30–39,…, 70–79 y old), gender, and rural versus urban location. The model is an extension of a model of myocardial infarction and stroke mortality developed by the Institute for Health Metrics and Evaluation [16]; however, the effect sizes in the model were updated using India-specific data from 2010 through 2012, as listed in the Text S1. The model simulates 10,000 individuals for each of the 24 age-, gender-, and location-specific cohorts. We then generate a “risk factor profile” for each individual in each cohort based on the WHO data listed in Tables S1, S2, S3, S4, S5, S6, S7, S8, S9, S10, S11, S12, which describes the population distribution of major CVD risk factors, correlations between the risk factors, and the relative risk of myocardial infarction and stroke mortality conferred by these risk factors. In other words, each of the 10,000 risk profiles for each cohort is different, based on age, gender, and location. Age was simulated as a continuous variable and then divided into cohorts. Age-, gender-, and location-specific secular trends in risk factor prevalence and mortality are included, based on the WHO Global InfoBase 2012 mortality database [17]. The 20 to 79-y-old age group was chosen because age-specific disaggregated data from India are available from which to specify their cardiovascular risks and tobacco use habits. The previous model [16] incorporated tobacco smoking, blood pressure, cholesterol, diabetes, and preexisting CVD and cerebrovascular disease, and was used to estimate worldwide CVD mortality rates; we modified the model to specifically include several different forms of tobacco use (specified further below) and capture the unique demographics and risks of the Indian population.

Six risk factors are incorporated into the risk factor profiles: systolic blood pressure, total cholesterol, tobacco use, diabetes, coronary heart disease, and cerebrovascular disease. Tobacco use is further subdivided into cigarette smoking, bidi (small hand-rolled cigarette) smoking, chewing tobacco, dual use (both chewing tobacco and smoking either bidis or cigarettes), secondhand smoke exposure, and former tobacco use. All of the data on risk factor prevalence, as well as on the impact of tobacco measures on cardiovascular risk, were specifically collected from population-representative, disaggregated surveys from India (Table 1). Body mass index was not included because, although it is correlated with blood pressure, cholesterol, and diabetes, it had a small effect size on myocardial infarctions or strokes when adjusting for the other risk factors [16].

**Table 1:** Model parameters and data sources.

Input	Specifications/Value	Source
Population size and secular trends	Breakdowns by age, gender, and urban/rural location	Indian census, 2011 [32]
Mortality rates from coronary heart disease, cerebrovascular disease, and other causes	See Tables S10, S11	India-specific WHO Global Burden of Disease estimates, 2008 [33]
Population distribution of systolic blood pressure	See Tables S1 and S8	India-specific WHO estimates, 2011 [11]
Population distribution of total cholesterol	See Tables S2 and S8	India-specific WHO estimates, 2012 [17]
Population distribution of tobacco use (further subdivided into passive exposure only, former user, cigarette smoking, bidi smoking, chewing tobacco, and dual use)	See Tables S3 and S8	India-specific data from the Global Adult Tobacco Survey, 2009–2010 [12]
Diabetes prevalence	See Tables S4 and S8	India-specific data from a cross-sectional survey, 2004 [34], updated using estimates from a Bayesian analysis of prevalence trends, 2011 [35]
Coronary heart disease prevalence	See Table S5 and S8	India-specific WHO estimates, 2010 [5]
Cerebrovascular disease prevalence	See Tables S6 and S8	India-specific WHO estimates, 2010 [18]
Correlation among risk factors listed above	See Table S7	India-specific data from the Institute for Health Metrics and Evaluation, 2007 [16]
Relative risk of coronary heart disease and cerebrovascular disease conferred by changes in each risk factor listed above	See Table S9	Prior reviews of international data [16],[36],[37]
Relative risk reduction of coronary heart disease and cerebrovascular disease conferred by aspirin, statin, and/or blood pressure treatments	See Table S12	Prior reviews of international data [16],[25]
Smoke-free laws: prohibit smoking in workplaces and public places	Reduces passive smoking probability by 64% (95% CI: 39%–89%) and active smoking probability for both bidis and cigarettes by 1% (0%–2%)	Systematic reviews and meta-analysis [22],[26],[38]
Brief cessation advice by health care providers: standardized motivational interviewing or cessation advice in primary and secondary care facilities	Reduces active smoking probability for both bidis and cigarettes by 1% (95% CI: 0%–3%)	Cochrane review [39]
Mass media campaign: anti-tobacco messaging covering channels of communication such as television, radio, newspapers, billboards, posters, leaflets	Reduces active smoking probability for both bidis and cigarettes by 5% (95% CI: 1%–11%)	Cochrane review and case study [23],[40]
Advertising ban: tobacco product advertising banned in films, sporting events, TV/radio, and print media	Reduces active smoking probability for both bidis and cigarettes by 6% (95% CI: 5%–7%)	National Bureau of Economic Research [20],[41]
Tax increases for bidis	50% tax reduces active bidi smoking probability by 4% (95% CI: 3%–5%), 300% tax reduces probability by 24% (95% CI: 22%–26%), and 500% tax reduces probability by 40% (95% CI: 37%–43%)	International Union Against Tuberculosis and Lung Disease [14]
Tax increases for cigarettes	50% tax increase reduces active cigarette smoking probability by 6% (95% CI: 3%–9%), 300% tax increase reduces probability by 31% (95% CI: 16%–47%), and 500% tax increase reduces probability by 52% (95% CI: 26%–78%)	International Union Against Tuberculosis and Lung Disease [14]

In each year of the simulation, an individual's probability of death from myocardial infarction, stroke, and all-cause mortality was calculated as a function of that individual's risk profile, the relative risk of disease conferred by each risk factor in their profile, and their age, gender, and location. The model's predictions of deaths were validated against Global Burden of Disease estimates for myocardial infarction, stroke, and other deaths for the years 2004 and 2008 (the two time points available) (Figure S1).

We ran the model 10,000 times using Monte Carlo sampling from the risk factor distributions in each cohort, the range of relative risk estimates used to estimate the probability of death for each individual, and demographic projection ranges from the Indian 2011 census to capture demographic uncertainty, generating 95% confidence intervals around the estimated mean rates of death from each cause in each cohort. This process included sampling repeatedly from the uncertainty distributions of the variables listed in Tables 1 and S1, S2, S3, S4, S5, S6, S7, S8, S9, S10, S11, S12. The model was implemented in MATLAB version R2012a (MathWorks).

Full details of the model, including complete equations and input data, appear in Text S1.

### Simulated Interventions

We simulated five tobacco control interventions specified in the FCTC: (1) smoke-free laws, (2) brief cessation advice by health providers, (3) mass media campaigns covering channels of communication such as television, radio, newspapers, billboards, posters, leaflets, or booklets intended to reach large numbers of people, (4) a tobacco advertising ban, and (5) increased cigarette and/or bidi taxes. The effect of each intervention on an individual's probability of tobacco use was taken from relevant international systematic reviews and meta-analyses, summarized in Table 1. We assumed that India's populace would react similarly to these interventions as other international populations; we chose meta-analyses that incorporated international settings to estimate intervention effects, as displayed in Table 1. We also developed a baseline simulation using rates of introduction of interventions based on real-world experiences [18]–[20], then performed several sensitivity analyses in which we varied the degrees of coverage to examine the impact of a range of possible implementation effects and timescales (Table 1). In our baseline simulation, we assumed that legislative interventions would be immediately introduced with full coverage throughout the country, but subsequent effects on smoking would occur linearly over a decade and further cardiovascular effects would also occur gradually over time, as detailed further below [21],[22]. In sensitivity analyses, we estimated the impact of a gradual introduction of legislation at a linear rate from 0% to 80% legislative coverage over 5 y, to achieve a steady state of 80% coverage, the level previously estimated as a target level for analogous interventions [16].

We varied the efficacy of each intervention around a normal distribution defined by the 95% confidence intervals given in Table 1 to characterize the uncertainty in our estimates. In the case of smoke-free laws, mass media campaigns, and a tobacco advertising ban, our sensitivity analysis included some low ranges of potential effectiveness or enforcement (Table 1) to simulate the potential outcomes when these measures are poorly enforced [18]–[20]. We also estimated that the CVD risk reduction benefits of quitting tobacco smoking would decline at an exponential rate over 10 y, with a time constant of 19.1 mo, yielding a 15% decline in risk in the first year, 36% by the end of 3 y, and the remaining benefits over the subsequent 8 y [21],[22].

In the case of taxation, we simulated cigarette and bidi taxation both individually and concurrently. The impact of taxation on consumption was calculated in a prior study that estimated the price elasticity of cigarette consumption (in which a 10% increase in cigarette prices reduced consumption by 3.4% in rural India and 1.9% in urban India) and bidi consumption (in which a 10% rise in bidi price reduced consumption by 9.2% in rural India and 8.5% in urban India) [14]. These estimates assume that all tax increases are passed on to customers, and do not account for possible tax evasion or smuggling. The WHO recommends that at least 70% of the price of a tobacco product be made up by tax, which would require an approximate 300% increase in the tax rate on cigarettes in India [23]. We therefore simulated this tax increase for cigarettes and bidis in our baseline simulation. In sensitivity analyses, we varied the taxation increase from a 50% to a 800% increase over existing tax rates. Taxation on bidis would have to increase approximately 800% to achieve the WHO recommendation.

### Independence and Synergy

In our baseline simulation, we forecast the impact of each intervention one at a time. We also simulated different degrees of synergy in the effectiveness of interventions. No studies have clearly established whether the same or different individuals would be prevented from tobacco use when these interventions are combined in India (e.g., if the effects of two interventions simultaneously may not be purely additive), although some evidence in other populations suggests that different tobacco control interventions may have synergy by enhancing the effectiveness of each intervention when multiple interventions are combined at the same time [24]. Hence, we conducted a sensitivity analysis in which we assumed no additive effect among interventions (i.e., the combined effect of multiple simultaneous interventions is equal to the effect of the intervention with the largest effect), an analysis where the effects are cumulative (i.e., the effect of multiple measures is equal to 1−[(1−risk reduction from intervention A)×(1−risk reduction from intervention B)×(1−risk reduction from intervention C)], etc.) and an analysis where we assumed that interventions reinforce each other synergistically by 25% (i.e., using the cumulative equation above, but with a 25% increase in the individual effectiveness shown in Table 1 of each intervention when the interventions are combined).

### Comparison with Pharmacological Interventions

We compared the effects of tobacco control interventions on myocardial infarction and stroke deaths with the effects of other interventions aimed at reducing cardiovascular risk, specifically the WHO's proposal to expand pharmacological treatment access to aspirin, antihypertensive medications, and statins. As described in Text S1, we sampled from normal distributions constructed from the 95% confidence intervals around the annual relative risk reduction for myocardial infarctions and stroke mortality conferred by these medications, and assumed their benefits were additive, as in prior simulations [16],[25]. We examined prior rates of expansion in access to these medications; penetration of treatment as primary prevention is below 50% in the UK National Health Service, for example, after over a decade of cardiovascular treatment guideline use [4],[26]. We simulated that, optimistically, 45% of the population in India that should receive treatment with these medications per WHO targets would have access to them over 10 y, paralleling the UK case. In sensitivity analyses, we also simulated the WHO target of achieving 80% treatment access linearly over 10 y (from current coverage estimates of 15%) [16]. The eligibility for medical treatment and relative risk reduction for myocardial infarction and stroke mortality from each type of medication (aspirin, antihypertensive, and statin treatment) is detailed in Text S1. We include treatment with an angiotensin-converting enzyme inhibitor and thiazide diuretic for those without CVD or cerebrovascular disease history, while including beta-blockers for those with such a history, as per WHO guidelines further detailed in Text S1 [16],[25]. As in prior simulations, we assumed that persons without a prior history of coronary heart disease or cerebrovascular disease would have 40% adherence to the medications (95% CI: 20%–60%, which we simulated by sampling from a normal distribution), while those with a coronary heart disease or cerebrovascular disease history would have 60% adherence (95% CI: 40%–80%, also sampled from a normal distribution) [16],[25]. We simulated the effects of these medications separately and jointly by assuming their effects were complementary and therefore additive.

## Results

### Scenario without Tobacco Control Measures

**Figure 1:**
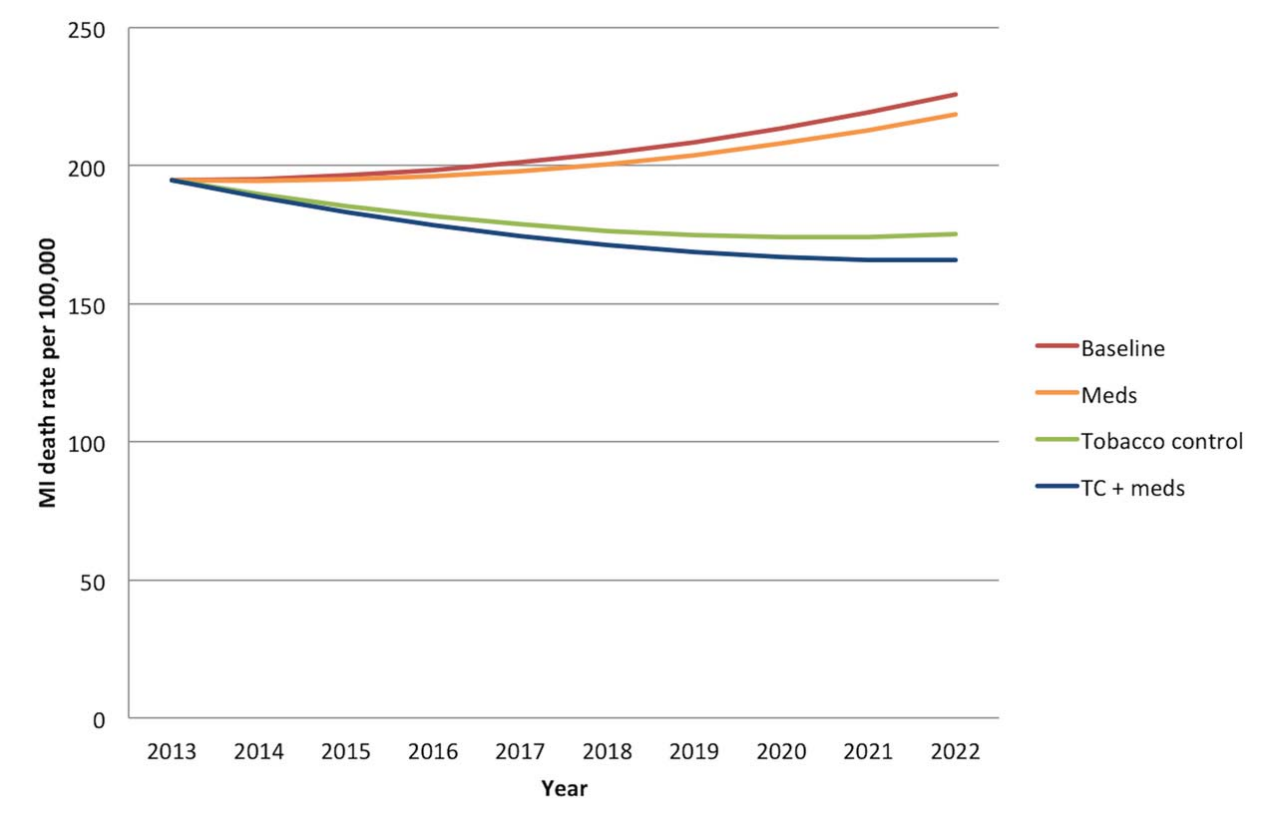
Overall mortality trend for myocardial infarctions in India over the period 2013–2022. “Meds” simulates the cumulative effects of aspirin, antihypertensive drugs, and statins. “Tobacco control” refers to a combination of smoke-free legislation, brief cessation advice by clinicians, a mass media campaign, a ban on advertising, and a 300% tax rate increase on both bidis and cigarettes with a cumulative impact equal to 1−([1−risk reduction from intervention A]×[1−risk reduction from intervention B], etc.). “TC+meds” refers to the combination of all medications and tobacco control measures, also assuming cumulative impact. MI, myocardial infarction.

**Figure 2:**
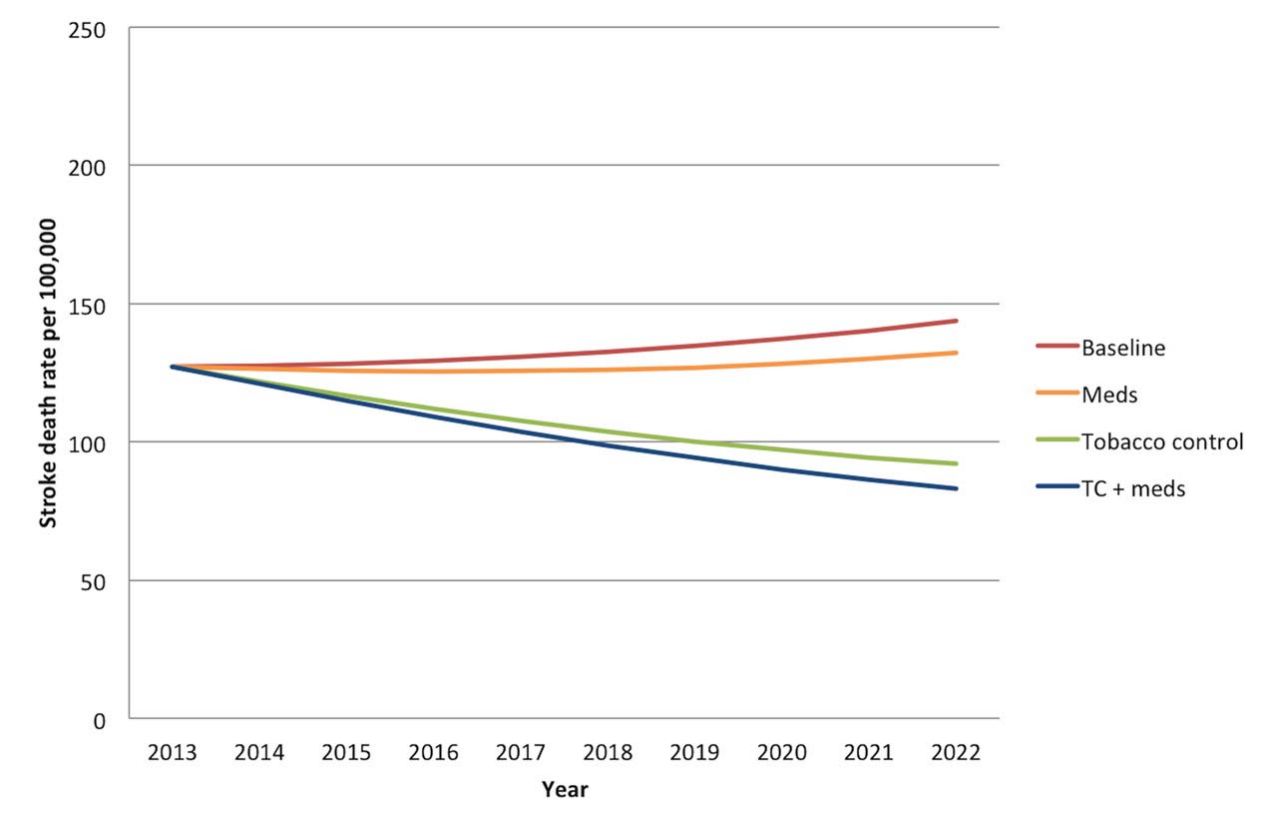
Overall mortality trend for strokes in India over the period 2013–2022. “Meds” simulates the cumulative effects of aspirin, antihypertensive drugs, and statins. “Tobacco control” refers to a combination of smoke-free legislation, brief cessation advice by clinicians, a mass media campaign, a ban on advertising, and a 300% tax rate increase on both bidis and cigarettes with a cumulative impact equal to 1−([1−risk reduction from intervention A]×[1−risk reduction from intervention B], etc.). “TC+meds” refers to the combination of all medications and tobacco control measures, also assuming cumulative impact.

Without any new interventions between 2013 and 2022, the 20- to 79-y-old population of India is projected to experience approximately 21.9 million (95% CI: 16.9 to 26.9 million) deaths from myocardial infarctions and 14.7 million (95% CI: 13.7 to 15.7 million) deaths from stroke, assuming the continuation of current trends in age-, gender-, and location-specific CVD risk factors and mortality rates. Figures 1 and 2 summarize the predicted overall mortality trend in cardiovascular deaths. Approximately 33% of the myocardial infarction deaths would be anticipated among urban men, followed by 31% among rural men, 19% among urban women, and 17% among rural women. About 27% of stroke deaths would be expected among urban men, 25% among rural men, 25% among urban women, and 23% among rural women.

### Comparative Effectiveness of Tobacco Control Measures

**Figure 3:**
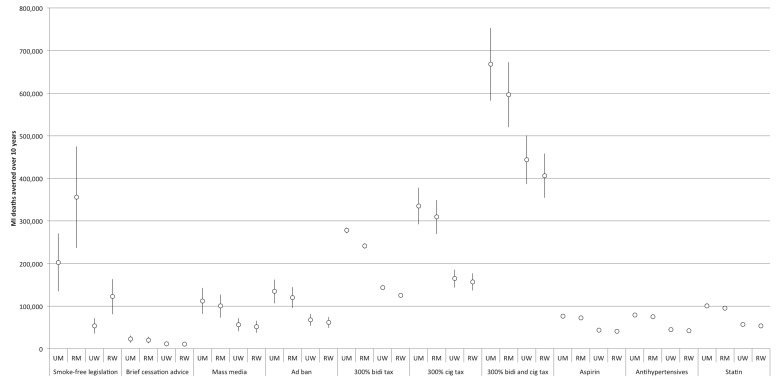
Comparative effectiveness of alternative tobacco control and pharmacological interventions on future myocardial infarction deaths, 2013–2022. 95% confidence intervals are displayed as error bars. MI, myocardial infarction; RM, rural men; RW, rural women; UM, urban men; UW, urban women.

**Figure 4:**
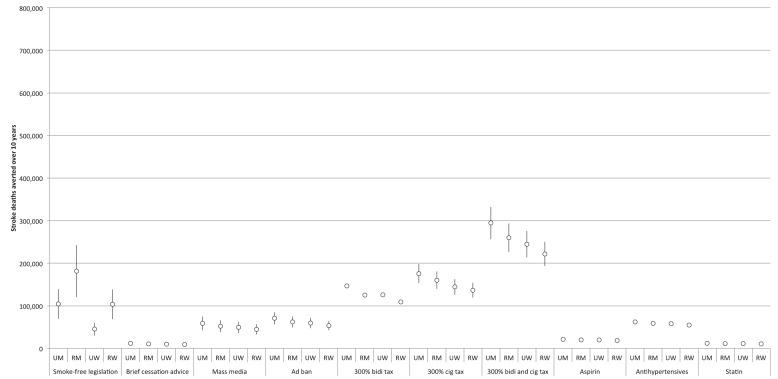
Comparative effectiveness of alternative tobacco control and pharmacological interventions on future stroke deaths, 2013–2022. 95% confidence intervals are displayed as error bars. RM, rural men; RW, rural women; UM, urban men; UW, urban women.

The comparative effectiveness of the simulated tobacco control interventions among different cohorts is illustrated in Figures 3 and 4. Table 2 provides details of the modeled absolute and percentage declines in myocardial infarction and stroke deaths attributable to each intervention over the decade from 2013 through 2022.

**Table 2:** Comparative effectiveness of tobacco control and pharmacological interventions in reducing myocardial infarction and stroke deaths, 2013–2022.

Category	Intervention	MI Deaths Averted (95% CI)	Percent Reduction in MI Deaths	Stroke deaths Averted (95% CI)	Percent Reduction in Stroke Deaths
**Tobacco control**	Smoke-free legislation	734,000 (487,000–981,000)	3.7	435,000 (289,000–581,000)	3.8
	Brief cessation advice	64,000 (40,000–88,000)	0.3	41,000 (25,000–57,000)	0.3
	Mass media	320,000 (233,000–407,000 )	1.6	205,000 (149,000–261,000)	0.3
	Advertising ban	384,000 (305,000–463,000)	1.9	246,000 (195,000–297,000)	2.0
	300% bidi tax	787,000 (768,000–806,000)	4.0	507,000 (495,000–519,000)	4.0
	300% cigarette tax	965,000 (841,000–1,089,000)	4.9	617,000 (538,000–696,000)	5.0
	300% bidi and cigarette tax	2,114,000 (1,843,000–2,385,000)	8.8	1,021,000 (890,000–1,152,000)	9.0
**Pharmacological**	Aspirin	232,000 (225,000–239,000)	1.1	79,000 (77,000–81,000)	0.5
	Anti-hypertensives	241,000 (230,000–252,000)	1.1	234,000 (224,000–244,000)	1.6
	Statin	307,000 (290,000–324,000)	1.4	44,000 (42,000–46,000)	0.3
**Combinations**	All meds	769,000 (677,000–861,000)	3.5	852,000 (751,000–953,000)	5.8
	All TC (no additive effects)	2,422,000 (1,501,000–3,343,000)	12.2	1,517,000 (940,000–2,094,000)	12.5
	All TC (cumulative effects)	5,018,000 (4,097,000–5,939,000)	25.3	3,182,000 (2,598,000–3,766,000)	25.8
	All TC (25% synergy)	6,272,000 (5,351,000–7,193,000)	31.6	3,978,000 (3,394,000–4,562,000)	32.3
	All TC+all meds	5,954,000 (5,033,000–6,875,000)	30.0	3,736,000 (3,158,000–4,314,000)	30.3

“All meds” assumes that the effects of aspirin, antihypertensive drugs, and statins are additive. “All TC” refers to a combination of smoke-free legislation, brief cessation advice by clinicians, a mass media campaign, a ban on advertising, and a 300% tax rate increase on both bidis and cigarettes. “No additive effects” means that only the impact of the most effective tobacco control intervention produces the resulting effectiveness of the tobacco control package. “Cumulative effects” assumes that a combined package of tobacco control interventions would have an impact equal to 1−([1−risk reduction from intervention A]×[1−risk reduction from intervention B], etc.). “25% synergy” assumes that when the interventions are combined, the impact of each individual intervention is amplified by 25%.

MI, myocardial infarction; TC, tobacco control.

The two most effective interventions were smoke-free legislation and tobacco taxation. Smoke-free legislation would be anticipated to avert approximately 0.7 million myocardial infarction deaths (95% CI: 0.4 to 1.0 million, about 3.7% of myocardial infarction deaths) and 0.4 million stroke deaths (95% CI: 0.3 to 0.6 million, 3.8%) over the decade. By comparison, a 300% increase in the cigarette tax rate would be anticipated to avert approximately 1.0 million myocardial infarction deaths (95% CI: 0.8 to 1.1 million, about 4.9% of myocardial infarction deaths) and 0.6 million stroke deaths (95% CI: 0.5 to 0.7 million, 5.0%) over the decade. A 300% increase in the bidi tax rate was not as effective as the cigarette tax increase; this bidi tax increase would avert approximately 0.8 million myocardial infarction deaths (95% CI: 0.7 to 0.9, about 4.0% of myocardial infarction deaths) and 0.5 million stroke deaths (95% CI: 0.4 to 0.6 million, 4.0%) over the decade. A 300% increase in both cigarette and bidi tax rates would be anticipated to avert approximately 2.1 million myocardial infarction deaths (95% CI: 1.8 to 2.4 million, about 8.8% of myocardial infarction deaths) and 1.0 million stroke deaths (95% CI: 0.9 to 1.2 million, 9.0%) over the decade.

The least effective population-level tobacco control intervention, as shown in Table 1, was brief cessation advice by health providers, which would be anticipated to avert approximately 60,000 myocardial infarction deaths (95% CI: 40,000 to 90,000, about 0.3% of myocardial infarction deaths) and 40,000 stroke deaths (95% CI: 30,000 to 60,000, about 0.3%) over the decade.

Nearly all of the tobacco control interventions affected urban men more than rural men, and urban women more than rural women. Between 28% and 35% of averted myocardial infarction deaths were among urban men, 28% to 48% among rural men, 7% to 21% among urban women, and 16% to 19% among rural women. Of the averted stroke deaths, 24% to 29% were among urban men, 25% to 42% among rural men, 10% to 24% among urban women, and 21% to 24% among rural women (see Figures 3 and 4 for breakdowns by intervention).

### Sensitivity Analysis of Tax Rates

We varied the rate of taxation of cigarettes and bidis both independently and together. An increase in the bidi tax rate by 800% would increase the number of myocardial infarctions averted from 0.8 to 2.0 million (95% CI: 1.97 to 2.06 million, 10.1% decrease) and the number of strokes averted from 0.5 to 1.3 million (95% CI: 1.27 to 1.32 million, 10.4%). An increase in the cigarette tax by 800% would similarly increase the number of myocardial infarctions averted from 1.0 to 2.5 million (95% CI: 2.2 to 2.8 million, 12.5% decrease) and the number of strokes averted from 0.6 to 1.6 million (95% CI: 1.4 to 1.8 million, 12.7%). Reducing the taxation rate to just a 50% increase above current levels caused a linear proportional drop in effectiveness, producing one-sixth the effect of the 300% tax increase.

### Potential Synergy

**Figure 5:**
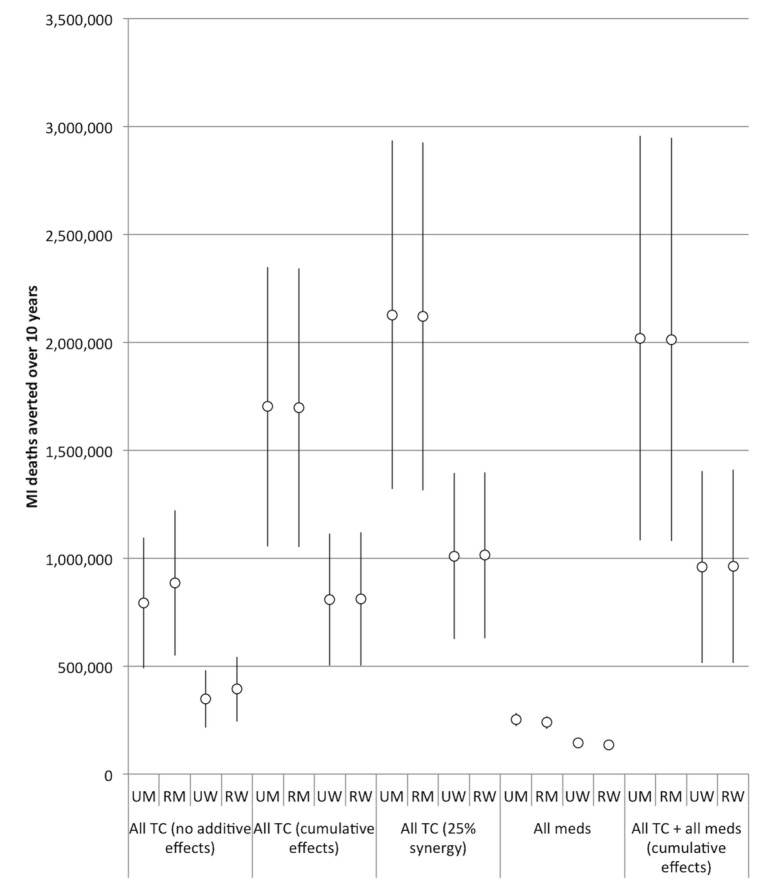
Impact of combining interventions on future myocardial infarction deaths, 2013–2022. Note the change in *y*-axis scale from Figure 2. “All meds” assumes the effects of aspirin, antihypertensive drugs, and statins are cumulative. “All TC” refers to a combination of smoke-free legislation, brief cessation advice by clinicians, a mass media campaign, a ban on advertising, and a 300% tax rate increase on both bidis and cigarettes. “No additive effects” means that only the impact of the most effective tobacco control intervention produces the resulting effectiveness of the tobacco control package. “Cumulative effects” assumes that a combined package of tobacco control interventions would have a cumulative impact equal to 1−([1−risk reduction from intervention A]×[1−risk reduction from intervention B], etc.). “25% synergy” assumes that when the interventions are combined cumulatively, the impact of each individual intervention is amplified by 25%. 95% confidence intervals are displayed as error bars. RM, rural men; RW, rural women; UM, urban men; UW, urban women.

**Figure 6:**
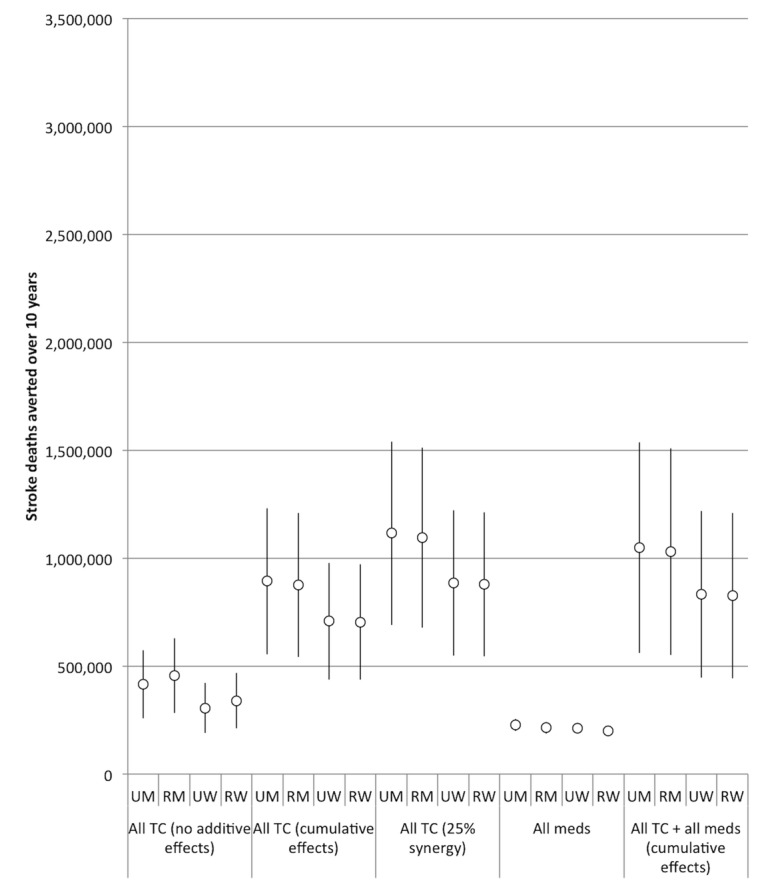
Impact of combining interventions on future stroke deaths, 2013–2022. Note the change in *y*-axis scale from Figure 2. “All meds” assumes the effects of aspirin, antihypertensive drugs, and statins are cumulative. “All TC” refers to a combination of smoke-free legislation, brief cessation advice by clinicians, a mass media campaign, a ban on advertising, and a 300% tax rate increase on both bidis and cigarettes. “No additive effects” means that only the impact of the most effective tobacco control intervention produces the resulting effectiveness of the tobacco control package. “Cumulative effects” assumes that a combined package of tobacco control interventions would have a cumulative impact equal to 1−([1−risk reduction from intervention A]×[1−risk reduction from intervention B], etc.). “25% synergy” assumes that when the interventions are combined cumulatively, the impact of each individual intervention is amplified by 25%. 95% confidence intervals are displayed as error bars. RM, rural men; RW, rural women; UM, urban men; UW, urban women.

Figures 5 and 6 illustrate the effects of different degrees of synergy between combined interventions. If we assume that tobacco control strategies that are combined will not have any additive effects (e.g., only the most potent control measure will have its effect exerted), then combining the five interventions (with the tax rate increase set to our baseline level of a 300% increase) would incorporate the passive smoking benefit reductions of smoke-free tobacco legislation and the active smoking benefit reductions of 300% increased tax rates on bidis and cigarettes. This scenario would be anticipated to avert approximately 2.4 million myocardial infarction deaths (95% CI: 1.5 to 3.3 million, 12.2% of myocardial infarction deaths) and 1.5 million stroke deaths (95% CI: 0.9 to 2.1 million, 12.5%) over the decade.

The estimated efficacy of the combined tobacco control program increased if we assumed cumulative benefits from the interventions (i.e., the effect of multiple measures is equal to 1−[(1−risk reduction from intervention A)×(1−risk reduction from intervention B)×(1−risk reduction from intervention C)], etc.), and increased further when we simulated a synergistic interaction (such that their combination enhances the efficacy of each individual intervention by 25%). However, as shown in Table 2 and Figures 5 and 6, there was a high degree of uncertainty in the simulations, given the compounded uncertainty of each individual intervention's impact. In the 25% synergy scenario, the tobacco control package of the five interventions would be anticipated to avert approximately 6.3 million myocardial infarction deaths (95% CI: 5.4 to 7.2 million, about 31.6% of myocardial infarction deaths) and 4.0 million stroke deaths (95% CI: 3.4 to 4.6 million, 32.3%) over the decade.

### Impact of Pharmacological Interventions

Figures 3 and 4 and Table 2 display results from our simulations of pharmacological therapies compared with tobacco control interventions. Statin therapy had the largest projected impact on myocardial infarction deaths among the medications (averting 1.4% of myocardial infarction deaths over the simulated period), while antihypertensive therapy had the greatest impact on stroke deaths (averting 1.6%).

If we assume cumulative benefits among the pharmacological therapies, then having all of the therapies available would be anticipated to avert approximately 0.8 million myocardial infarction deaths (95% CI: 0.7 to 0.9 million, about 3.5% of myocardial infarction deaths) and 0.9 million stroke deaths (95% CI: 0.8 to 1.0 million, 5.8%) over the decade. This indicates that the combination of medications was about as effective as the 300% bidi tax rate increase alone in preventing myocardial infarction deaths and slightly more effective than the 300% cigarette tax increase alone in preventing stroke deaths, though the medication combination is not as effective as the combination of 300% tax increases on both cigarettes and bidis (Table 2). The combined effects of all the pharmacological interventions were also smaller than the effect of implementing smoke-free laws. Note that these scenarios assume a slow rate of increase of access to pharmacological therapies.

The impact of medications was, however, only moderately sensitive to the rate of diffusion in the population. If 80% coverage were achieved by 10 y, then the combination of therapies (assuming additive benefits between the medications) would be anticipated to avert approximately 1.0 million myocardial infarction deaths (95% CI: 0.9 to 1.2 million, about 4.7% of myocardial infarction deaths) and 1.0 million stroke deaths (95% CI: 0.9 to 1.1 million, 6.8%) over the decade. In this scenario, too, the combination of medications would be less effective than the 300% tax rate increase on both bidis and cigarettes.

### Combining Tobacco Control with Pharmacological Interventions

Adding a tobacco control package (including smoke-free legislation, brief cessation advice, a mass media campaign, advertising ban, and 300% tax rate increase on both cigarettes and bidis) to the combination of all of the pharmacological interventions, assuming all interventions have cumulative effects, we would anticipate averting approximately 6.0 million myocardial infarction deaths (95% CI: 5.0 to 6.9 million, about 30.0% of myocardial infarction deaths) and 3.7 million stroke deaths (95% CI: 3.2 to 4.3 million, 30.3%) per year over the decade (Table 2). This combination of additive tobacco control and all three types of medications was slightly less powerful than the full tobacco control package alone with the assumption that 25% synergy occurred between the tobacco control interventions.

### Impact among Different Age Groups

Without any new interventions, the largest burden of deaths from myocardial infarctions and strokes would be expected to accrue in the 60- to 69-y-old age group more than in any other age cohort; this age group would experience 48% of myocardial infarction deaths and 55% of stroke deaths. When tobacco control measures are instituted, the greatest benefits similarly accrue in the 60- to 69-y-old age group, who make up 46% of averted myocardial infarction deaths and 54% of averted stroke deaths from any of the tobacco control interventions except smoke-free legislation. None of the tobacco control interventions differed in how they affected different age groups, except for smoke-free legislation, which—because of differential exposure to secondhand smoke among different age groups—had an increased benefit to younger populations. Of the myocardial infarctions averted by smoke-free legislation, 41% would be expected to accrue among 60- to 69-y-olds, and 12% would be averted in the 30- to 39-y-old age group, as opposed to only 9% from any of the other interventions. Of the strokes averted by smoke-free legislation, 50% would be expected to accrue among 60- to 69-y-olds, and 11% would be averted in the 30- to 39-y-old age group, as opposed to only 8% from any of the other interventions. The pharmacological interventions were most beneficial to the 60- to 69-y-old cohort; this age group accounted for 48% of the myocardial infarction deaths averted by medicines and 55% of the strokes averted by medicines.

### Speed of Implementation

Our baseline simulation was constructed to reflect the speed of implementation observed in other developing countries [12]. If tobacco control measures are delayed such that they are introduced at a linear rate from 0% to 80% coverage over 5 y, and thereafter remain at a steady state of 80% coverage, their impact over the next decade could be lowered by approximately 60% from the above estimates (95% CI: 45%–70%). The slower rate of implementation would result in a shift in the gender distribution of deaths as well. Because trends in death disfavor males, of the total deaths taking place over the next decade, the proportion of deaths made up by males would be expected to be 13 percentage points higher than in the baseline scenario of 64% male deaths.

## Discussion

Substantial evidence links tobacco use to CVD, yet tobacco use in India and several other LMICs is on the rise [6]. This worsening trend led the UN High Level Meeting on Prevention and Control of Non-Communicable Diseases to recommend that countries accelerate implementation of the FCTC. Our findings indicate that full implementation of key FCTC articles in India would yield substantial reductions in mortality from myocardial infarctions and stroke, despite projected increases in other risk factors for CVD such as hypertension and diabetes. Far from achieving “diminishing returns,” vigorous implementation of these tobacco control policies would be expected to avert 25% of all predicted CVD deaths, equivalent to over 9 million averted deaths, over the decade 2013 and 2022 under a reasonable set of modeling assumptions.

Furthermore, the population-level benefits of implementing strong tobacco control policies were five times greater than a similarly aggressive program to implement pharmacological interventions (Figure 3), assuming that India's implementation of pharmacological therapy is no faster than in high-income nations like the UK. However, critically, we would anticipate additive benefits of implementing pharmacological interventions concurrently with strong tobacco control policies: the combined package of both pharmacological and tobacco control interventions would not be redundant, and could avert nearly a third of cardiovascular and cerebrovascular mortality over the next decade by tackling multiple risk factors for myocardial infarctions and strokes.

Among the interventions we simulated, smoke-free legislation and tax increases on both cigarettes and bidis were the most effective at the population level. Smoke-free legislation, an advertising ban, and a mass media campaign had wide confidence intervals because their degree of population effectiveness was subject to the degree of effective enforcement.

We used, to our knowledge for the first time, population-representative data to represent the co-morbid risks of CVD among both urban and rural populations in India, and among both men and women in multiple agegroups. We found that nearly all sectors of Indian society were likely to benefit from both tobacco control and pharmacological therapies. However, the populations benefiting most were urban males and persons in the 60- to 69-y-old age category. This is due to the higher baseline prevalence of co-morbid risk factors among urban males and this older age group; hence, these populations achieve greater risk reduction from the simulated interventions.

### Limitations

Projections from mathematical models such as this one are limited by assumptions and uncertainties inherent to the exercise of mathematical modeling. Models are useful at a policy level for comparing different population-wide interventions because it is, for practical purposes, challenging to institute large-scale randomized trials of such population-level interventions for comparing the potential future implications of such interventions when several demographic and biological factors come into play. We modeled the effects of tobacco control in accordance with published international data and assumed that the Indian population would respond to tobacco control measures similarly to other populations. While this model was validated against independent Global Burden of Disease estimates, such estimates are not themselves a real population registry, but rather are largely imputed data from sporadic surveys; hence, this model cannot be thought of as calibrated or validated retrospectively against a real population. A potential amendment to this problem is prospective validation and further refinement of this model against emerging datasets that will provide some further information on risks, such as the WHO Study on Global Ageing and Adult Health [11].

These estimates serve as an important template for other LMICs looking for effective policies to stem the growing tide of noncommunicable diseases, as India's tobacco use, as well as the prevalence of other major risk factors, is thought to be representative of many other middle-income nations [11]. We simulated several alternative additive or multiplicative effects of different tobacco control approaches, but we do not yet know whether some interventions are more effective in certain age groups or in one gender over the other—for example, there are potentially more significant declines in smoking when interventions target youth [27]. We also simulated the impact of pharmacological therapy using optimistic medication adherence rates, but if true adherence rates are lower, this would further reinforce our findings of relatively higher and faster impact from tobacco control interventions. Furthermore, we did not simulate the cost-effectiveness of the various interventions, as the cost estimates are currently inconsistent, in part because of rapidly changing prices of drugs and the unclear administrative costs of many of the control measures described, particularly in rural zones [28]–[30]. Nevertheless, prior models based on reasonable estimates of cost have found tobacco control measures and pharmacological interventions to be highly cost-effective in LMICs [15],[25]. It is nevertheless notable that smoke-free measures and tax increases come with relatively low administrative costs, unlike pharmacological interventions.

Our estimates of the risk factor prevalence and mortality rates among cohorts defined by age, gender, and location were based on estimates from the WHO, and there is uncertainty about these estimates as they are subject to surveillance bias that may reduce the estimated mortality rates of women, rural populations, or the isolated elderly; however, sensitivity and uncertainty analyses around our input parameters suggest that our primary findings are not changed despite wide variations in these data estimates. We modeled only the short-term effects of reduced tobacco use on CVD. It has been suggested that lack of smoke exposure over longer terms will produce greater benefits to cardiovascular health [31]; such an assumption would result in larger reductions in CVD from tobacco control than we present here. It is also important to note, given the smoking options in India, that an increase in the tax rate on just cigarettes, but not bidis, also may lead to increased smoking of bidis, which could reduce the beneficial impact of the cigarette tax. Furthermore, the data we have available on India's specific CVD mortality rates and risk factor prevalence levels do not explicitly disaggregate the population by socioeconomic status, only by age, gender, and urban/rural location. Understanding socioeconomic differences should be a high priority for future research. Finally, we did not model many other emerging interventions that could affect the risk factors simulated here, such as nutritional interventions.

### Conclusion

Given the complexities of India's tobacco and CVD epidemics, it is important to understand how heterogeneities within the large Indian population may affect both the risk of disease as well as the impact of various policy and health care interventions. In this study, we provide to our knowledge the first model that incorporates population-representative data from India disaggregated by age, gender, and location for all of the major CVD risk factors as well as for specific types of tobacco use. Prior models have either used “average” Indian or regional disease rates, have not captured various types of tobacco use other than smoking, or have not incorporated the multiple risk factors affecting CVD in addition to tobacco use. This means that, for the first time, we can study some health disparities in CVD and in tobacco use within the large and varied Indian population, as well as heterogeneity in the impact of proposed interventions. These results provide clear justification for India's Ministry of Health and Family Welfare to engage in greater enforcement of the FCTC and the Indian legislation that enacts the FCTC in the country, the Cigarettes and Other Tobacco Products Act.

Our simulations suggest that the implementation of recommended tobacco control interventions in India would yield substantial and rapid health benefits, but those benefits may accumulate most among males, urban dwellers, and older adults. Effective implementation of FCTC provisions remains a major challenge in India. At present, smoke-free legislation in India is not comprehensive and is poorly enforced [12]. There is little indication that brief cessation advice is routinely administered, and additional resources may be required to strengthen implementation. Tobacco taxes in India would also need to be substantially increased and harmonized between different tobacco products to comply with WHO recommendations, and achieve desired population-level disease reductions [14],[15]. Cigarettes are currently taxed according to their physical length in India, meaning that uniform tax increases will encourage product substitution unless large price differentials between products are addressed.

Optimizing preventive interventions for CVD remains a significant challenge for developing countries like India. Our model demonstrates synergies between tobacco control and pharmacological therapies for key CVD risk factors. It does not, however, support the idea that enhanced prevalence of key risk factors renders diminished results from tobacco control interventions. Rather, policymakers should take note that fuller and faster implementation of existing FCTC provisions would likely be a substantial boon to efforts to reduce CVD mortality in India and other LMICs.

## Supporting Information

Figure S1 **Face validity of the model against historical data from 2004 and 2008, when inputting year 2000 data into the model.** These results are from the full model including tobacco use.
(DOCX)

Table S1 **Population distribution of systolic blood pressure.**
(DOCX)

Table S2 **Population distribution of total cholesterol.**
(DOCX)

Table S3 **Population distribution of tobacco exposure.**
(DOCX)

Table S4 **Diabetes prevalence.**
(DOCX)

Table S5 **Coronary heart disease prevalence.**
(DOCX)

Table S6 **Cerebrovascular disease prevalence.**
(DOCX)

Table S7 **Correlation matrix among risk factors described in Tables S1, S2, S3, S4, S5, S6.**
(DOCX)

Table S8 **Secular trends in risk factor levels (percent change in prevalence per year).**
(DOCX)

Table S9 **Relative risk per unit increase in each risk factor.**
(DOCX)

Table S10 **Mortality rates from coronary heart disease, cerebrovascular disease, and other causes (per year).**
(DOCX)

Table S11 **Secular trends in mortality rates (percent change in mortality rate per year).**
(DOCX)

Table S12 **Annual relative risk reduction for myocardial infarctions and strokes from each type of medication (aspirin for coronary/cerebrovascular disease, statin treatment for hyperlipidemia, and blood pressure treatment for hypertension).**
(DOCX)

Text S1 **Model description.**
(DOCX)

## Abbreviations

CVD: cardiovascular disease
FCTC: Framework Convention on Tobacco Control
LMICs: low- and middle-income countries
WHO: World Health Organization
