# Supplementary material for: The Effect of Tobacco Control Measures during a Period of Rising Cardiovascular Disease Risk in India: A Mathematical Model of Myocardial Infarction and Stroke
Source: PLoS Med. 2013 Jul 9;10(7):e1001480. doi: 10.1371/journal.pmed.1001480 (PMC3706364; doi:10.1371/journal.pmed.1001480)
Supplement: Figure S1 — Face validity of the model against historical data from 2004 and 2008, when inputting year 2000 data into the model. These results are from the full model including tobacco use. (DOCX) [file pmed.1001480.s001.docx]

# Figure S1. Face validity of the model against historical data from 2004 and 2008, when inputting year 2000 data into the model . These results are from the full model including tobacco use.

Data are from independent WHO estimates ([3](#_ENREF_3)).
