# Supplementary material for: The Effect of Tobacco Control Measures during a Period of Rising Cardiovascular Disease Risk in India: A Mathematical Model of Myocardial Infarction and Stroke
Source: PLoS Med. 2013 Jul 9;10(7):e1001480. doi: 10.1371/journal.pmed.1001480 (PMC3706364; doi:10.1371/journal.pmed.1001480)
Supplement: Table S1 — Population distribution of systolic blood pressure. (DOCX) [file pmed.1001480.s002.docx]

# Table S1: Population distribution of systolic blood pressure

| Age (years) | Male urban | | Female urban | | Male rural | | Female rural | |
| --- | --- | --- | --- | --- | --- | --- | --- | --- |
|  | Mean | SD | Mean | SD | Mean | SD | Mean | SD |
| 20-29 | 119.3 | 13.1 | 111.4 | 13.8 | 110.1 | 13.3 | 110.2 | 14.0 |
| 30-39 | 121.9 | 20.3 | 114.3 | 16.3 | 112.5 | 19.5 | 113.1 | 16.0 |
| 40-49 | 121.0 | 24.3 | 118.6 | 17.1 | 111.6 | 22.5 | 117.4 | 17.0 |
| 50-59 | 125.2 | 21.0 | 123.6 | 15.2 | 115.4 | 20.8 | 122.4 | 15.1 |
| 60-69 | 127.2 | 17.7 | 128.0 | 12.1 | 117.4 | 17.8 | 126.6 | 12.2 |
| 70-79 | 130.5 | 23.8 | 129.9 | 12.6 | 120.3 | 22.7 | 128.5 | 12.5 |

# Systolic blood pressure is described in mmHg from a population-representative study across Indian districts. SD: standard deviation. For all SI Tables, estimates are given for the year 2013, and for subsequent years the secular trends listed in SI Table 8 are applied. Source: ([5](#_ENREF_5)).

# 
