# Supplementary material for: The Effect of Tobacco Control Measures during a Period of Rising Cardiovascular Disease Risk in India: A Mathematical Model of Myocardial Infarction and Stroke
Source: PLoS Med. 2013 Jul 9;10(7):e1001480. doi: 10.1371/journal.pmed.1001480 (PMC3706364; doi:10.1371/journal.pmed.1001480)
Supplement: Table S2 — Population distribution of total cholesterol. (DOCX) [file pmed.1001480.s003.docx]

# Table S2: Population distribution of total cholesterol

| Age (years) | Male urban | | Female urban | | Male rural | | Female rural | |
| --- | --- | --- | --- | --- | --- | --- | --- | --- |
|  | Mean | SD | Mean | SD | Mean | SD | Mean | SD |
| 20-29 | 5.45 | 1.01 | 5.33 | 0.96 | 4.82 | 0.93 | 4.70 | 0.52 |
| 30-39 | 5.51 | 1.01 | 5.46 | 1.00 | 5.08 | 0.92 | 4.89 | 0.66 |
| 40-49 | 5.67 | 1.06 | 5.75 | 1.12 | 5.13 | 0.74 | 5.10 | 0.89 |
| 50-59 | 5.80 | 1.15 | 5.94 | 1.15 | 4.80 | 0.52 | 4.96 | 0.75 |
| 60-69 | 5.81 | 1.19 | 5.97 | 1.09 | 4.68 | 0.47 | 4.81 | 0.52 |
| 70-79 | 5.81 | 1.19 | 5.97 | 1.09 | 4.68 | 0.47 | 4.81 | 0.52 |

# Total cholesterol is described in mmol/L from a study across Indian districts. SD: standard deviation. For all SI Tables, estimates are given for the year 2013, and for subsequent years the secular trends listed in SI Table 8 are applied. Source: ([3](#_ENREF_3)).

# 
