# Supplementary material for: The Effect of Tobacco Control Measures during a Period of Rising Cardiovascular Disease Risk in India: A Mathematical Model of Myocardial Infarction and Stroke
Source: PLoS Med. 2013 Jul 9;10(7):e1001480. doi: 10.1371/journal.pmed.1001480 (PMC3706364; doi:10.1371/journal.pmed.1001480)
Supplement: Table S3 — Population distribution of tobacco exposure. (DOCX) [file pmed.1001480.s004.docx]

# Table S3: Population distribution of tobacco exposure

Tobacco exposure is a prevalence rate, further subdivided into multiple types of exposure. Of those exposed, we categorize exposures into: passive exposure only, former user, cigarette smoking, bidi smoking, and dual use (chewing and any type of smoking). These prevalence rates are 2013 estimates based on updates to a population-representative survey of adults in Indian districts ([6](#_ENREF_6)). SD: Standard deviation. For all SI Tables, estimates are given for the year 2013, and for subsequent years the secular trends listed in SI Table 8 are applied.

A) Exposed to any form of tobacco:

| Age (years) | Male urban | | Female urban | | Male rural | | Female rural | |
| --- | --- | --- | --- | --- | --- | --- | --- | --- |
|  | Mean | SD | Mean | SD | Mean | SD | Mean | SD |
| 20-29 | 65.2% | 16.4% | 51.1% | 24.6% | 74.1% | 21.2% | 55.4% | 20.8% |
| 30-39 | 71.2% | 19.0% | 53.7% | 22.2% | 83.2% | 29.0% | 60.3% | 18.2% |
| 40-49 | 73.7% | 18.0% | 56.4% | 20.2% | 84.5% | 27.9% | 68.9% | 19.9% |
| 50-59 | 74.3% | 16.6% | 58.2% | 18.8% | 86.2% | 26.2% | 67.4% | 17.1% |
| 60-69 | 73.9% | 12.4% | 60.4% | 17.8% | 86.3% | 24.6% | 72.1% | 19.9% |
| 70-79 | 77.2% | 10.1% | 60.1% | 18.3% | 94.3% | 25.9% | 71.4% | 20.7% |

B) Proportion exposed who have passive tobacco exposure only:

| Age (years) | Male urban | | Female urban | | Male rural | | Female rural | |
| --- | --- | --- | --- | --- | --- | --- | --- | --- |
|  | Mean | SD | Mean | SD | Mean | SD | Mean | SD |
| 20-29 | 49.2% | 8.1% | 88.5% | 21.7% | 32.2% | 6.8% | 74.3% | 15.5% |
| 30-39 | 37.4% | 7.1% | 79.7% | 17.7% | 18.7% | 5.4% | 60.7% | 11.0% |
| 40-49 | 32.9% | 5.9% | 71.5% | 14.4% | 16.9% | 4.7% | 41.6% | 8.3% |
| 50-59 | 32.0% | 5.3% | 66.2% | 12.4% | 14.8% | 3.9% | 44.7% | 7.7% |
| 60-69 | 32.6% | 4.0% | 60.4% | 10.8% | 14.6% | 3.6% | 35.7% | 7.1% |
| 70-79 | 27.3% | 2.7% | 61.2% | 11.2% | 5.6% | 1.5% | 37.1% | 7.7% |

C) Proportion exposed who are former tobacco users:

| Age (years) | Male urban | | Female urban | | Male rural | | Female rural | |
| --- | --- | --- | --- | --- | --- | --- | --- | --- |
|  | Mean | SD | Mean | SD | Mean | SD | Mean | SD |
| 20-29 | 4.4% | 0.7% | 0.0% | 0.0% | 5.4% | 1.1% | 0.5% | 0.1% |
| 30-39 | 4.8% | 0.9% | 0.3% | 0.1% | 8.2% | 2.4% | 0.9% | 0.2% |
| 40-49 | 9.2% | 1.6% | 0.5% | 0.1% | 11.8% | 3.3% | 1.2% | 0.2% |
| 50-59 | 11.6% | 1.9% | 1.8% | 0.3% | 16.7% | 4.4% | 4.2% | 0.7% |
| 60-69 | 17.0% | 2.1% | 1.9% | 0.3% | 19.2% | 4.7% | 4.7% | 0.9% |
| 70-79 | 24.4% | 2.5% | 0.9% | 0.2% | 33.8% | 8.8% | 2.7% | 0.5% |

D) Proportion exposed who are cigarette smokers only:

| Age (years) | Male urban | | Female urban | | Male rural | | Female rural | |
| --- | --- | --- | --- | --- | --- | --- | --- | --- |
|  | Mean | SD | Mean | SD | Mean | SD | Mean | SD |
| 20-29 | 3.7% | 0.6% | 0.00% | 0.00% | 1.2% | 0.3% | 0.02% | 0.00% |
| 30-39 | 7.2% | 1.4% | 0.00% | 0.00% | 2.7% | 0.8% | 0.00% | 0.00% |
| 40-49 | 9.3% | 1.7% | 0.02% | 0.00% | 2.6% | 0.7% | 0.19% | 0.04% |
| 50-59 | 8.5% | 1.4% | 0.03% | 0.01% | 3.0% | 0.8% | 0.01% | 0.00% |
| 60-69 | 5.9% | 0.7% | 0.03% | 0.01% | 0.8% | 0.2% | 0.01% | 0.00% |
| 70-79 | 5.6% | 0.6% | 0.03% | 0.01% | 0.7% | 0.2% | 0.01% | 0.00% |

E) Proportion exposed who are bidi smokers only:

| Age (years) | Male urban | | Female urban | | Male rural | | Female rural | |
| --- | --- | --- | --- | --- | --- | --- | --- | --- |
|  | Mean | SD | Mean | SD | Mean | SD | Mean | SD |
| 20-29 | 1.8% | 0.3% | 0.1% | 0.0% | 4.2% | 0.9% | 0.5% | 0.1% |
| 30-39 | 5.9% | 1.1% | 0.3% | 0.1% | 8.6% | 2.5% | 1.2% | 0.2% |
| 40-49 | 9.3% | 1.7% | 0.9% | 0.2% | 16.3% | 4.5% | 2.2% | 0.4% |
| 50-59 | 12.2% | 2.0% | 1.2% | 0.2% | 19.6% | 5.1% | 3.5% | 0.6% |
| 60-69 | 10.6% | 1.3% | 1.3% | 0.2% | 15.2% | 3.7% | 4.3% | 0.9% |
| 70-79 | 10.1% | 1.0% | 1.3% | 0.2% | 13.9% | 3.6% | 4.4% | 0.9% |

F) Proportion exposed who are tobacco chewers only:

| Age (years) | Male urban | | Female urban | | Male rural | | Female rural | |
| --- | --- | --- | --- | --- | --- | --- | --- | --- |
|  | Mean | SD | Mean | SD | Mean | SD | Mean | SD |
| 20-29 | 31.5% | 5.2% | 11.0% | 2.7% | 44.5% | 9.4% | 23.9% | 5.0% |
| 30-39 | 33.4% | 6.4% | 19.1% | 4.2% | 44.5% | 12.9% | 35.5% | 6.5% |
| 40-49 | 28.4% | 5.1% | 26.0% | 5.2% | 36.3% | 10.1% | 52.7% | 10.5% |
| 50-59 | 25.3% | 4.2% | 29.4% | 5.5% | 32.9% | 8.6% | 44.3% | 7.6% |
| 60-69 | 26.6% | 3.3% | 34.8% | 6.2% | 37.1% | 9.1% | 49.4% | 9.8% |
| 70-79 | 25.5% | 2.6% | 34.9% | 6.4% | 33.9% | 8.8% | 50.0% | 10.3% |

G) Proportion exposed who are dual users (chewing and smoking):

| Age (years) | Male urban | | Female urban | | Male rural | | Female rural | |
| --- | --- | --- | --- | --- | --- | --- | --- | --- |
|  | Mean | SD | Mean | SD | Mean | SD | Mean | SD |
| 20-29 | 9.4% | 1.5% | 0.4% | 0.1% | 12.5% | 2.6% | 0.7% | 0.2% |
| 30-39 | 11.3% | 2.2% | 0.5% | 0.1% | 17.2% | 5.0% | 1.8% | 0.3% |
| 40-49 | 10.9% | 2.0% | 1.2% | 0.2% | 16.0% | 4.5% | 2.1% | 0.4% |
| 50-59 | 10.5% | 1.7% | 1.3% | 0.2% | 13.1% | 3.4% | 3.4% | 0.6% |
| 60-69 | 7.4% | 0.9% | 1.6% | 0.3% | 13.2% | 3.2% | 5.9% | 1.2% |
| 70-79 | 7.1% | 0.7% | 1.6% | 0.3% | 12.1% | 3.1% | 5.9% | 1.2% |

# 
