# Supplementary material for: The Effect of Tobacco Control Measures during a Period of Rising Cardiovascular Disease Risk in India: A Mathematical Model of Myocardial Infarction and Stroke
Source: PLoS Med. 2013 Jul 9;10(7):e1001480. doi: 10.1371/journal.pmed.1001480 (PMC3706364; doi:10.1371/journal.pmed.1001480)
Supplement: Table S4 — Diabetes prevalence. (DOCX) [file pmed.1001480.s005.docx]

# Table S4: Diabetes prevalence

| Age (years) | Male urban | | Female urban | | Male rural | | Female rural | |
| --- | --- | --- | --- | --- | --- | --- | --- | --- |
|  | Mean | SD | Mean | SD | Mean | SD | Mean | SD |
| 20-29 | 5.5% | 1.1% | 1.2% | 1.1% | 3.8% | 0.9% | 0.8% | 0.9% |
| 30-39 | 10.6% | 1.1% | 2.2% | 1.1% | 4.2% | 0.9% | 0.9% | 0.9% |
| 40-49 | 14.0% | 1.1% | 2.9% | 1.1% | 6.4% | 0.9% | 1.3% | 0.9% |
| 50-59 | 14.8% | 1.1% | 3.1% | 1.1% | 6.0% | 0.9% | 1.3% | 0.9% |
| 60-69 | 18.2% | 1.1% | 3.8% | 1.1% | 15.7% | 0.9% | 3.3% | 0.9% |
| 70-79 | 18.2% | 1.1% | 3.8% | 1.1% | 15.7% | 0.9% | 3.3% | 0.9% |

# Diabetes prevalence is estimated from a random multistage cross-sectional population survey ([7](#_ENREF_7)), updated to 2013 using secular trend estimates from a Bayesian analysis of diabetes prevalence trends ([8](#_ENREF_8)). SD: standard deviation. For all SI Tables, estimates are given for the year 2013, and for subsequent years the secular trends listed in SI Table 8 are applied.

# 
