# Supplementary material for: The Effect of Tobacco Control Measures during a Period of Rising Cardiovascular Disease Risk in India: A Mathematical Model of Myocardial Infarction and Stroke
Source: PLoS Med. 2013 Jul 9;10(7):e1001480. doi: 10.1371/journal.pmed.1001480 (PMC3706364; doi:10.1371/journal.pmed.1001480)
Supplement: Table S5 — Coronary heart disease prevalence. (DOCX) [file pmed.1001480.s006.docx]

# Table S5: Coronary heart disease prevalence

| Age (years) | Male urban | | Female urban | | Male rural | | Female rural | |
| --- | --- | --- | --- | --- | --- | --- | --- | --- |
|  | Mean | SD | Mean | SD | Mean | SD | Mean | SD |
| 20-29 | 1.38% | 0.82% | 1.65% | 1.37% | 1.56% | 0.27% | 1.24% | 0.28% |
| 30-39 | 3.01% | 1.85% | 3.57% | 1.80% | 1.56% | 0.45% | 1.34% | 0.37% |
| 40-49 | 6.53% | 2.55% | 8.56% | 2.79% | 1.93% | 0.20% | 3.10% | 1.10% |
| 50-59 | 12.78% | 4.91% | 13.23% | 2.89% | 2.89% | 0.31% | 5.04% | 0.07% |
| 60-69 | 17.37% | 6.67% | 17.54% | 3.83% | 7.11% | 0.77% | 6.74% | 0.10% |
| 70-79 | 17.37% | 6.67% | 17.54% | 3.83% | 7.11% | 0.77% | 6.74% | 0.10% |

# Coronary heart disease prevalence is from a prior WHO meta-analysis of Indian district surveys ([9](#_ENREF_9)), updated to the year 2013 based on WHO estimates of secular trends ([3](#_ENREF_3)). SD: standard deviation. For all SI Tables, estimates are given for the year 2013, and for subsequent years the secular trends listed in SI Table 8 are applied.

# 
