# Supplementary material for: The Effect of Tobacco Control Measures during a Period of Rising Cardiovascular Disease Risk in India: A Mathematical Model of Myocardial Infarction and Stroke
Source: PLoS Med. 2013 Jul 9;10(7):e1001480. doi: 10.1371/journal.pmed.1001480 (PMC3706364; doi:10.1371/journal.pmed.1001480)
Supplement: Table S6 — Cerebrovascular disease prevalence. (DOCX) [file pmed.1001480.s007.docx]

# Table S6: Cerebrovascular disease prevalence

| Age (years) | Male urban | | Female urban | | Male rural | | Female rural | |
| --- | --- | --- | --- | --- | --- | --- | --- | --- |
|  | Mean | SD | Mean | SD | Mean | SD | Mean | SD |
| 20-29 | 0.00% | 0.00% | 0.02% | 0.51% | 0.00% | 0.00% | 0.02% | 0.51% |
| 30-39 | 0.00% | 0.00% | 0.02% | 0.02% | 0.00% | 0.00% | 0.02% | 0.02% |
| 40-49 | 0.05% | 0.32% | 0.05% | 0.32% | 0.05% | 0.32% | 0.05% | 0.32% |
| 50-59 | 0.58% | 0.42% | 0.58% | 0.42% | 0.58% | 0.42% | 0.58% | 0.42% |
| 60-69 | 0.88% | 0.18% | 0.88% | 0.18% | 0.88% | 0.18% | 0.88% | 0.18% |
| 70-79 | 0.91% | 0.02% | 0.91% | 0.02% | 0.91% | 0.02% | 0.91% | 0.02% |

# Cerebrovascular disease prevalence is from a prior WHO meta-analysis of Indian district surveys ([9](#_ENREF_9)), updated to the year 2013 based on WHO estimates of secular trends ([3](#_ENREF_3)). SD: standard deviation. For all SI Tables, estimates are given for the year 2013, and for subsequent years the secular trends listed in SI Table 8 are applied.

# 
