# Supplementary material for: The Effect of Tobacco Control Measures during a Period of Rising Cardiovascular Disease Risk in India: A Mathematical Model of Myocardial Infarction and Stroke
Source: PLoS Med. 2013 Jul 9;10(7):e1001480. doi: 10.1371/journal.pmed.1001480 (PMC3706364; doi:10.1371/journal.pmed.1001480)
Supplement: Table S7 — Correlation matrix among risk factors described in Tables S1, S2, S3, S4, S5, S6. (DOCX) [file pmed.1001480.s008.docx]

# Table S7: Correlation matrix among risk factors described in SI Tables 1-6

|  | Systolic blood pressure | Cholesterol | Tobacco exposure | Diabetes | Coronary heart disease | Cerebrovascular disease |
| --- | --- | --- | --- | --- | --- | --- |
| Systolic blood pressure | 1.000 | 0.174 | -0.096 | 0.087 | 0.037 | 0.045 |
| Cholesterol | 0.174 | 1.000 | -0.107 | 0.098 | 0.014 | 0.012 |
| Tobacco exposure | -0.096 | -0.107 | 1.000 | -0.034 | -0.003 | -0.003 |
| Diabetes | 0.087 | 0.098 | -0.034 | 1.000 | 0.037 | 0.031 |
| Coronary heart disease | 0.037 | 0.014 | -0.003 | 0.037 | 1.000 | 0.200 |
| Cerebrovascular disease | 0.045 | 0.012 | -0.003 | 0.031 | 0.200 | 1.000 |

# Correlation coefficients between risk factors in the model, as provided by the Institute for Health Metrics and Evaluation from a prior assessment of risk factors based on population surveys ([1](#_ENREF_1)).

# 
