# Supplementary material for: The Effect of Tobacco Control Measures during a Period of Rising Cardiovascular Disease Risk in India: A Mathematical Model of Myocardial Infarction and Stroke
Source: PLoS Med. 2013 Jul 9;10(7):e1001480. doi: 10.1371/journal.pmed.1001480 (PMC3706364; doi:10.1371/journal.pmed.1001480)
Supplement: Table S8 — Secular trends in risk factor levels (percent change in prevalence per year). (DOCX) [file pmed.1001480.s009.docx]

# Table S8: Secular trends in risk factor levels (% change in prevalence per year) These are relative increases in the prevalence rate (not absolute increases).

| Age | Gender | Location | SBP | Chol | Tob exp | DM | IHD | Stroke |
| --- | --- | --- | --- | --- | --- | --- | --- | --- |
| 20-29 | Male | Urban | 0.42% | 0.21% | -0.01% | 0.17% | 0.05% | 0.00% |
| 30-39 | Male | Urban | 0.58% | 0.07% | -0.02% | 0.23% | 0.05% | 0.00% |
| 40-49 | Male | Urban | 0.32% | 0.04% | 0.00% | 0.30% | 0.03% | -0.27% |
| 50-59 | Male | Urban | 0.29% | 0.06% | 0.02% | 0.07% | 0.04% | -0.01% |
| 60-69 | Male | Urban | 0.29% | 0.07% | 0.11% | 0.29% | 0.05% | 0.01% |
| 70-79 | Male | Urban | 0.29% | 0.07% | -0.50% | 0.15% | 0.04% | -0.03% |
| 20-29 | Male | Rural | 0.42% | 0.21% | -0.01% | 0.17% | 0.03% | 0.00% |
| 30-39 | Male | Rural | 0.58% | 0.07% | -0.02% | 0.23% | 0.05% | 0.00% |
| 40-49 | Male | Rural | 0.32% | 0.04% | 0.00% | 0.30% | 0.02% | -0.27% |
| 50-59 | Male | Rural | 0.29% | 0.06% | 0.02% | 0.07% | 0.02% | -0.01% |
| 60-69 | Male | Rural | 0.29% | 0.07% | 0.11% | 0.29% | 0.01% | 0.01% |
| 70-79 | Male | Rural | 0.29% | 0.07% | -0.50% | 0.15% | 0.03% | -0.03% |
| 20-29 | Female | Urban | 0.28% | 0.25% | -0.15% | 1.00% | 0.05% | -0.18% |
| 30-39 | Female | Urban | 0.48% | 0.18% | -0.19% | 0.37% | 0.05% | -0.47% |
| 40-49 | Female | Urban | 0.25% | 0.08% | -0.01% | 0.66% | 0.02% | -0.11% |
| 50-59 | Female | Urban | 0.18% | 0.21% | 0.22% | 0.09% | 0.01% | 0.00% |
| 60-69 | Female | Urban | 0.20% | 0.13% | 0.19% | 0.16% | 0.05% | 0.00% |
| 70-79 | Female | Urban | 0.20% | 0.13% | 0.19% | 0.07% | 0.04% | 0.05% |
| 20-29 | Female | Rural | 0.28% | 0.25% | -0.15% | 1.00% | 0.04% | -0.18% |
| 30-39 | Female | Rural | 0.48% | 0.18% | -0.19% | 0.37% | 0.05% | -0.47% |
| 40-49 | Female | Rural | 0.25% | 0.08% | -0.01% | 0.66% | 0.03% | -0.11% |
| 50-59 | Female | Rural | 0.18% | 0.21% | 0.22% | 0.09% | 0.04% | 0.00% |
| 60-69 | Female | Rural | 0.20% | 0.13% | 0.19% | 0.16% | 0.04% | 0.00% |
| 70-79 | Female | Rural | 0.20% | 0.13% | 0.19% | 0.07% | 0.03% | 0.05% |

# Numbers listed describe percentage change in prevalence rates per year in each cohort from the sources described in SI Tables 1-7. SBP = systolic blood pressure; Chol = total cholesterol; Tob exp = tobacco exposure; DM = diabetes; CHD = coronary heart disease; Stroke = cerebrovascular disease. Sources: ([3](#_ENREF_3), [5-7](#_ENREF_5), [9](#_ENREF_9)).
