# Supplementary material for: The Effect of Tobacco Control Measures during a Period of Rising Cardiovascular Disease Risk in India: A Mathematical Model of Myocardial Infarction and Stroke
Source: PLoS Med. 2013 Jul 9;10(7):e1001480. doi: 10.1371/journal.pmed.1001480 (PMC3706364; doi:10.1371/journal.pmed.1001480)
Supplement: Table S9 — Relative risk per unit increase in each risk factor. (DOCX) [file pmed.1001480.s010.docx]

# Table S9: Relative risk per unit increase in each risk factor

Estimates are from a prior series of meta-analyses using international data ([1](#_ENREF_1), [10](#_ENREF_10), [11](#_ENREF_11)). A “unit increase” is defined as a 1mmHg increase for systolic blood pressure, a 1mmol/L increase in total cholesterol, and for the dichotomous variables (any kind of tobacco use and diabetes), a unit increase is defined as going from not exposed to exposed (e.g., becoming newly diabetic, or newly starting tobacco smoking).

# of coronary heart disease

| Age | Gender | Location | SBP | Chol | Passive | Former | Cigs | Bidis | Chew | Dual | DM |
| --- | --- | --- | --- | --- | --- | --- | --- | --- | --- | --- | --- |
| 20-29 | Male | Urban | 1.08 | 3.65 | 2.05 | 2.06 | 2.43 | 2.42 | 2.27 | 2.60 | 2.03 |
| 30-39 | Male | Urban | 1.07 | 3.65 | 2.05 | 2.06 | 2.43 | 2.42 | 2.27 | 2.60 | 2.03 |
| 40-49 | Male | Urban | 1.06 | 2.08 | 2.05 | 2.06 | 2.43 | 2.42 | 2.27 | 2.60 | 2.03 |
| 50-59 | Male | Urban | 1.05 | 1.55 | 1.62 | 1.62 | 1.84 | 1.84 | 1.75 | 1.94 | 2.03 |
| 60-69 | Male | Urban | 1.03 | 1.42 | 1.52 | 1.52 | 1.70 | 1.70 | 1.62 | 1.78 | 2.03 |
| 70-79 | Male | Urban | 1.02 | 1.42 | 1.28 | 1.28 | 1.38 | 1.38 | 1.34 | 1.43 | 2.03 |
| 20-29 | Male | Rural | 1.08 | 3.65 | 2.05 | 2.06 | 2.43 | 2.42 | 2.27 | 2.60 | 2.03 |
| 30-39 | Male | Rural | 1.07 | 3.65 | 2.05 | 2.06 | 2.43 | 2.42 | 2.27 | 2.60 | 2.03 |
| 40-49 | Male | Rural | 1.06 | 2.08 | 2.05 | 2.06 | 2.43 | 2.42 | 2.27 | 2.60 | 2.03 |
| 50-59 | Male | Rural | 1.05 | 1.55 | 1.62 | 1.62 | 1.84 | 1.84 | 1.75 | 1.94 | 2.03 |
| 60-69 | Male | Rural | 1.03 | 1.42 | 1.52 | 1.52 | 1.70 | 1.70 | 1.62 | 1.78 | 2.03 |
| 70-79 | Male | Rural | 1.02 | 1.42 | 1.28 | 1.28 | 1.38 | 1.38 | 1.34 | 1.43 | 2.03 |
| 20-29 | Female | Urban | 1.08 | 3.65 | 1.87 | 1.87 | 2.18 | 2.18 | 2.05 | 2.32 | 2.54 |
| 30-39 | Female | Urban | 1.07 | 3.65 | 1.87 | 1.87 | 2.18 | 2.18 | 2.05 | 2.32 | 2.54 |
| 40-49 | Female | Urban | 1.06 | 2.08 | 1.87 | 1.87 | 2.18 | 2.18 | 2.05 | 2.32 | 2.54 |
| 50-59 | Female | Urban | 1.05 | 1.55 | 1.82 | 1.83 | 2.12 | 2.12 | 2.00 | 2.26 | 2.54 |
| 60-69 | Female | Urban | 1.03 | 1.42 | 1.52 | 1.52 | 1.70 | 1.70 | 1.62 | 1.78 | 2.54 |
| 70-79 | Female | Urban | 1.02 | 1.42 | 1.23 | 1.23 | 1.31 | 1.31 | 1.28 | 1.35 | 2.54 |
| 20-29 | Female | Rural | 1.08 | 3.65 | 1.87 | 1.87 | 2.18 | 2.18 | 2.05 | 2.32 | 2.54 |
| 30-39 | Female | Rural | 1.07 | 3.65 | 1.87 | 1.87 | 2.18 | 2.18 | 2.05 | 2.32 | 2.54 |
| 40-49 | Female | Rural | 1.06 | 2.08 | 1.87 | 1.87 | 2.18 | 2.18 | 2.05 | 2.32 | 2.54 |
| 50-59 | Female | Rural | 1.05 | 1.55 | 1.82 | 1.83 | 2.12 | 2.12 | 2.00 | 2.26 | 2.54 |
| 60-69 | Female | Rural | 1.03 | 1.42 | 1.52 | 1.52 | 1.70 | 1.70 | 1.62 | 1.78 | 2.54 |
| 70-79 | Female | Rural | 1.02 | 1.42 | 1.23 | 1.23 | 1.31 | 1.31 | 1.28 | 1.35 | 2.54 |

# SBP = systolic blood pressure; Chol = total cholesterol; Passive = passive tobacco exposure; Former = former tobacco use; Cigs = cigarette smoking; Bidis = bidi smoking; Chew = tobacco chewing; Dual = dual use (chewing and smoking tobacco); DM = diabetes. For dichotomous variables (e.g., diabetes), relative risk refers to the risk increase in converting from 0 (not diabetic) to 1 (diabetic).

# (B) of cerebrovascular disease

| Age | Gender | Location | SBP | Chol | Passive | Former | Cigs | Bidis | Chew | Dual | DM |
| --- | --- | --- | --- | --- | --- | --- | --- | --- | --- | --- | --- |
| 20-29 | Male | Urban | 1.10 | 1.48 | 2.05 | 2.06 | 2.43 | 2.42 | 2.27 | 2.60 | 2.00 |
| 30-39 | Male | Urban | 1.09 | 1.35 | 2.05 | 2.06 | 2.43 | 2.42 | 2.27 | 2.60 | 2.00 |
| 40-49 | Male | Urban | 1.08 | 1.42 | 2.05 | 2.06 | 2.43 | 2.42 | 2.27 | 2.60 | 2.00 |
| 50-59 | Male | Urban | 1.07 | 1.35 | 1.62 | 1.62 | 1.84 | 1.84 | 1.75 | 1.94 | 2.00 |
| 60-69 | Male | Urban | 1.05 | 1.25 | 1.52 | 1.52 | 1.70 | 1.70 | 1.62 | 1.78 | 2.00 |
| 70-79 | Male | Urban | 1.03 | 1.09 | 1.28 | 1.28 | 1.38 | 1.38 | 1.34 | 1.43 | 2.00 |
| 20-29 | Male | Rural | 1.10 | 1.48 | 2.05 | 2.06 | 2.43 | 2.42 | 2.27 | 2.60 | 2.00 |
| 30-39 | Male | Rural | 1.09 | 1.35 | 2.05 | 2.06 | 2.43 | 2.42 | 2.27 | 2.60 | 2.00 |
| 40-49 | Male | Rural | 1.08 | 1.42 | 2.05 | 2.06 | 2.43 | 2.42 | 2.27 | 2.60 | 2.00 |
| 50-59 | Male | Rural | 1.07 | 1.35 | 1.62 | 1.62 | 1.84 | 1.84 | 1.75 | 1.94 | 2.00 |
| 60-69 | Male | Rural | 1.05 | 1.25 | 1.52 | 1.52 | 1.70 | 1.70 | 1.62 | 1.78 | 2.00 |
| 70-79 | Male | Rural | 1.03 | 1.09 | 1.28 | 1.28 | 1.38 | 1.38 | 1.34 | 1.43 | 2.00 |
| 20-29 | Female | Urban | 1.10 | 1.48 | 1.87 | 1.87 | 2.18 | 2.18 | 2.05 | 2.32 | 2.04 |
| 30-39 | Female | Urban | 1.09 | 1.35 | 1.87 | 1.87 | 2.18 | 2.18 | 2.05 | 2.32 | 2.04 |
| 40-49 | Female | Urban | 1.08 | 1.42 | 1.87 | 1.87 | 2.18 | 2.18 | 2.05 | 2.32 | 2.04 |
| 50-59 | Female | Urban | 1.07 | 1.35 | 1.82 | 1.83 | 2.12 | 2.12 | 2.00 | 2.26 | 2.04 |
| 60-69 | Female | Urban | 1.05 | 1.25 | 1.52 | 1.52 | 1.70 | 1.70 | 1.62 | 1.78 | 2.04 |
| 70-79 | Female | Urban | 1.03 | 1.09 | 1.23 | 1.23 | 1.31 | 1.31 | 1.28 | 1.35 | 2.04 |
| 20-29 | Female | Rural | 1.10 | 1.48 | 1.87 | 1.87 | 2.18 | 2.18 | 2.05 | 2.32 | 2.04 |
| 30-39 | Female | Rural | 1.09 | 1.35 | 1.87 | 1.87 | 2.18 | 2.18 | 2.05 | 2.32 | 2.04 |
| 40-49 | Female | Rural | 1.08 | 1.42 | 1.87 | 1.87 | 2.18 | 2.18 | 2.05 | 2.32 | 2.04 |
| 50-59 | Female | Rural | 1.07 | 1.35 | 1.82 | 1.83 | 2.12 | 2.12 | 2.00 | 2.26 | 2.04 |
| 60-69 | Female | Rural | 1.05 | 1.25 | 1.52 | 1.52 | 1.70 | 1.70 | 1.62 | 1.78 | 2.04 |
| 70-79 | Female | Rural | 1.03 | 1.09 | 1.23 | 1.23 | 1.31 | 1.31 | 1.28 | 1.35 | 2.04 |

# SBP = systolic blood pressure; Chol = total cholesterol; Passive = passive tobacco exposure; Former = former tobacco use; Cigs = cigarette smoking; Bidis = bidi smoking; Chew = tobacco chewing; Dual = dual use (chewing and smoking tobacco); DM = diabetes. For dichotomous variables (e.g., diabetes), relative risk refers to the risk increase in converting from 0 (not diabetic) to 1 (diabetic).
