# Supplementary material for: The Effect of Tobacco Control Measures during a Period of Rising Cardiovascular Disease Risk in India: A Mathematical Model of Myocardial Infarction and Stroke
Source: PLoS Med. 2013 Jul 9;10(7):e1001480. doi: 10.1371/journal.pmed.1001480 (PMC3706364; doi:10.1371/journal.pmed.1001480)
Supplement: Table S10 — Mortality rates from coronary heart disease, cerebrovascular disease, and other causes (per year). (DOCX) [file pmed.1001480.s011.docx]

# Table S10: Mortality rates from coronary heart disease, cerebrovascular disease, and other causes (per year). The denominator is total population (this is % of population per year dying from each cause).

| Age | Gender | Location | Heart | Cerebrovascular | Other |
| --- | --- | --- | --- | --- | --- |
| 20-29 | Male | Urban | 0.137% | 0.115% | 0.235% |
| 30-39 | Male | Urban | 0.137% | 0.115% | 0.235% |
| 40-49 | Male | Urban | 0.286% | 0.486% | 3.071% |
| 50-59 | Male | Urban | 0.286% | 0.486% | 3.071% |
| 60-69 | Male | Urban | 0.435% | 0.857% | 5.907% |
| 70-79 | Male | Urban | 0.435% | 0.857% | 5.907% |
| 20-29 | Male | Rural | 0.137% | 0.115% | 0.235% |
| 30-39 | Male | Rural | 0.137% | 0.115% | 0.235% |
| 40-49 | Male | Rural | 0.286% | 0.486% | 3.071% |
| 50-59 | Male | Rural | 0.286% | 0.486% | 3.071% |
| 60-69 | Male | Rural | 0.435% | 0.857% | 5.907% |
| 70-79 | Male | Rural | 0.435% | 0.857% | 5.907% |
| 20-29 | Female | Urban | 0.009% | 0.154% | 0.123% |
| 30-39 | Female | Urban | 0.009% | 0.154% | 0.123% |
| 40-49 | Female | Urban | 0.053% | 0.498% | 2.540% |
| 50-59 | Female | Urban | 0.053% | 0.498% | 2.540% |
| 60-69 | Female | Urban | 0.097% | 0.842% | 4.957% |
| 70-79 | Female | Urban | 0.097% | 0.842% | 4.957% |
| 20-29 | Female | Rural | 0.009% | 0.154% | 0.123% |
| 30-39 | Female | Rural | 0.009% | 0.154% | 0.123% |
| 40-49 | Female | Rural | 0.053% | 0.498% | 2.540% |
| 50-59 | Female | Rural | 0.053% | 0.498% | 2.540% |
| 60-69 | Female | Rural | 0.097% | 0.842% | 4.957% |
| 70-79 | Female | Rural | 0.097% | 0.842% | 4.957% |

# Mortality rates are from World Health Organization Global Burden of Disease estimates ([12](#_ENREF_12)). For all SI Tables, estimates are given for the year 2013, and for subsequent years the secular trends listed in SI Table 11 are applied.
