# Supplementary material for: The Effect of Tobacco Control Measures during a Period of Rising Cardiovascular Disease Risk in India: A Mathematical Model of Myocardial Infarction and Stroke
Source: PLoS Med. 2013 Jul 9;10(7):e1001480. doi: 10.1371/journal.pmed.1001480 (PMC3706364; doi:10.1371/journal.pmed.1001480)
Supplement: Table S11 — Secular trends in mortality rates (percent change in mortality rate per year). (DOCX) [file pmed.1001480.s012.docx]

# Table S11: Secular trends in mortality rates (percent change in mortality rate per year)

| Age | Gender | Location | Heart | Cerebrovascular | Other |
| --- | --- | --- | --- | --- | --- |
| 20-29 | Male | Urban | 2.7% | 8.0% | -2.5% |
| 30-39 | Male | Urban | 2.7% | 8.0% | -2.5% |
| 40-49 | Male | Urban | -0.9% | 4.3% | -0.3% |
| 50-59 | Male | Urban | -0.9% | 4.3% | -0.3% |
| 60-69 | Male | Urban | -4.5% | 0.6% | 2.0% |
| 70-79 | Male | Urban | -4.5% | 0.6% | 2.0% |
| 20-29 | Male | Rural | 2.7% | 8.0% | -2.5% |
| 30-39 | Male | Rural | 2.7% | 8.0% | -2.5% |
| 40-49 | Male | Rural | -0.9% | 4.3% | -0.3% |
| 50-59 | Male | Rural | -0.9% | 4.3% | -0.3% |
| 60-69 | Male | Rural | -4.5% | 0.6% | 2.0% |
| 70-79 | Male | Rural | -4.5% | 0.6% | 2.0% |
| 20-29 | Female | Urban | -5.6% | 6.4% | -1.0% |
| 30-39 | Female | Urban | -5.6% | 6.4% | -1.0% |
| 40-49 | Female | Urban | -7.0% | 12.1% | -3.9% |
| 50-59 | Female | Urban | -7.0% | 12.1% | -3.9% |
| 60-69 | Female | Urban | -8.5% | 0.6% | 1.9% |
| 70-79 | Female | Urban | -8.5% | 0.6% | 1.9% |
| 20-29 | Female | Rural | -5.6% | 6.4% | -1.0% |
| 30-39 | Female | Rural | -5.6% | 6.4% | -1.0% |
| 40-49 | Female | Rural | -7.0% | 12.1% | -3.9% |
| 50-59 | Female | Rural | -7.0% | 12.1% | -3.9% |
| 60-69 | Female | Rural | -8.5% | 0.6% | 1.9% |
| 70-79 | Female | Rural | -8.5% | 0.6% | 1.9% |
