# Supplementary material for: The Effect of Tobacco Control Measures during a Period of Rising Cardiovascular Disease Risk in India: A Mathematical Model of Myocardial Infarction and Stroke
Source: PLoS Med. 2013 Jul 9;10(7):e1001480. doi: 10.1371/journal.pmed.1001480 (PMC3706364; doi:10.1371/journal.pmed.1001480)
Supplement: Table S12 — Annual relative risk reduction for myocardial infarctions and strokes from each type of medication (aspirin for coronary/cerebrovascular disease, statin treatment for hyperlipidemia, and blood pressure treatment for hypertension). (DOCX) [file pmed.1001480.s013.docx]

# Table S12: Annual relative risk reduction for myocardial infarctions and strokes from each type of medication (aspirin for coronary/cerebrovascular disease, statin treatment for hyperlipidemia and blood pressure treatment for hypertension)

| Indication | Drug | MI risk | Stroke risk |
| --- | --- | --- | --- |
| No cardiovascular or cerebrovascular disease (primary prevention) | Statin | 0.69 (0.59-0.79) | 0.94 (0.78-1.14) |
|  | BP meds (ACEI and/or thiazide) | 0.66 (0.6-0.77) | 0.51 (0.45-0.58) |
|  | Aspirin | 0.68 (0.60–0.77) | 0.84 (0.75–0.93) |
| Cardiovascular or cerebrovascular disease (secondary prevention) | Statin | 0.71 (0.62-0.82) | 0.81 (0.66-1) |
|  | BP meds (ACEI and/or beta-blocker) | 0.8 (0.7-0.9) | 0.68 (0.56-0.84) |
|  | Aspirin | 0.66 (0.6–0.72) | 0.78 (0.72–0.84) |

Estimates are based upon prior reviews of international data ([1](#_ENREF_1), [13](#_ENREF_13)). Primary prevention with statin was applied to those treated for hyperlipidemia (defined as total cholesterol > 5.2 mmol/L) and/or known cardiovascular disease; blood pressure treatment was applied to those with hypertension (defined as blood pressure >140/90 mmHg) and those with known cardiovascular disease; and aspirin was applied to those with known cardiovascular disease, diabetic men over 50 and diabetic women over 60, and men over 45 and women over 55 with an absolute risk of a cardiovascular event of >25% over 10 years. Rates of access to medical treatment and estimates of adherence are detailed in the main text.
