# Supplementary material for: The Effect of Tobacco Control Measures during a Period of Rising Cardiovascular Disease Risk in India: A Mathematical Model of Myocardial Infarction and Stroke
Source: PLoS Med. 2013 Jul 9;10(7):e1001480. doi: 10.1371/journal.pmed.1001480 (PMC3706364; doi:10.1371/journal.pmed.1001480)
Supplement: Text S1 — Model description. (DOCX) [file pmed.1001480.s014.docx]

# Text S1.

# Model description

## Overview

The model used in this study is a discrete-time microsimulation model, which simulates myocardial infarction and stroke events and deaths at the level of the individual. The model creates a series of individual histories for members of each population cohort being studied, spanning the time frame from the year 2013 to the year 2023. Unlike a typical Markov model, this microsimulation model can capture the impact of individual-level interventions on individual risk factor profiles, not just the average population effect of an intervention—allowing for complex relationships among multiple risk factors and interventions to be incorporated into the experiment.

The modeling calculations employed here were previously devised by the Institute of Health Metrics and Evaluation for simulations of overall worldwide cardiovascular disease rates and the impact of pharmacological therapies ([1](#_ENREF_1)). This prior model did include tobacco smoking as a risk factor, but not other forms of tobacco use, and did not assess tobacco control measures. Here, we expand the model to multiple forms of tobacco use and use the same calculation approach to construct a model specific to the Indian population to simulate tobacco control interventions. We provide a review of the calculation approach and input data here in sufficient detail for replication of our results. The full model will be made available as open-source code in the international BioModels Database (<http://www.ebi.ac.uk/biomodels-main/>) concurrent with publication.

We begin the simulation by generating ten thousand individuals for each of 24 cohorts, where each cohort is defined by age (categorized into 10-year age clusters from 20-29 through 70-79 years old, with exact age distributed within the cluster according to Indian Census demography estimates as of 2011 ([2](#_ENREF_2))), gender, and location (urban or rural). To account for people graduating from one cohort to the next, we keep track of the individual age of simulated individuals and apply age-specific risks of heart disease and stroke as detailed below. To account for demographic shifts over the 10 years, we also account for how individuals enter the youngest cohort (20-29 years old) and leave all cohorts (mortality) at different rates based on their age, gender and urban or rural location; these rates of entry and exit have been calculated and projected into the future by the Indian census ([2](#_ENREF_2)) and World Health Organization ([12](#_ENREF_12)), and are detailed further below. Individual-level risk factor profiles for each of the 10,000 individuals in each cohort consist of systolic blood pressure, total cholesterol, whether the person has passive tobacco exposure (secondhand smoke), former tobacco use, current cigarette smoking, current bidi smoking, current use of chewing tobacco, dual use of both chewing and smoking tobacco in any form diabetes, coronary heart disease and cerebrovascular disease. As described in detail below, these risk profiles are generated using Monte-Carlo sampling from the distributions of risk factors for each cohort listed in SI Tables 1 through 6 (from the World Health Organization, WHO ([5](#_ENREF_5))), using the correlation matrix between the risk factors described by SI Table 7 (from the Institute for Health Metrics and Evaluation ([1](#_ENREF_1))) and adjusting each risk factor in each year of the simulation to reflect secular changes in risk (e.g., to capture how the risk of tobacco smoking is increasing at a given rate per year among some cohorts) (SI Table 8) ([3](#_ENREF_3)). Details on how these calculations are performed are provided below.

## Risk factor profiles

To generate each individual’s risk factor profile, a random number *r* is sampled from a normal distribution of mean 0 and standard deviation 1. For each continuous risk factor *i* (systolic blood pressure, total cholesterol), the individual’s risk factor value (i.e., their individual systolic blood pressure in mmHg or total cholesterol in mmol/L) is determined by the following function form, which was found to correct for the right-skewed distributions of these risk factors in the prior IHME analysis ([1](#_ENREF_1)):

(1) $x_{i}=e^{(r\sigma_{i}+\mu_{i})}$

where *x* is the continuous risk factor value (e.g., the systolic blood pressure) for the individual for risk factor *i*, *σ* is the transformed standard deviation of the risk factor in the individual’s cohort that year, and *μ* is the transformed mean value of the risk factor in the individual’s cohort that year. The variable *σ* is multiplied by *r* to transform the sampled random normal distribution (mean 0 and standard deviation 1) to the standard deviation of the risk factor, then added to *μ* to shift the mean of the distribution to the risk factor’s mean value. Transformations are used to correct for the right-skewed nature of the risk factor distributions. The transformations, derived previously ([1](#_ENREF_1)), are as follows:

(2) $\mu_{i}=\ln(\omega_{i}^{2})-\frac{\ln(\omega_{i}^{2}+\delta_{i}^{2})}{2}$

and

(3) $\sigma_{i}=\ln\sqrt{\ln(\delta_{i}^{2})+e^{2\ln(\omega_{i})}}-2\ln(\omega_{i})$

where *ω* is the mean and *δ* is the standard deviation of risk factor *i'*s distribution for the individual’s cohort that year.

For dichotomous risk factors (passive tobacco exposure, former tobacco use, current cigarette smoking, current bidi smoking, current use of chewing tobacco, dual use, diabetes, previous ischemic heart disease, and previous cerebrovascular disease), an individual is assigned to have that risk factor with a probability *r* equal to the prevalence of the risk factor in the individual’s cohort that year. The main text Table 1 lists how these prevalence rates were adjusted for each tobacco control scenario based on systematic reviews and meta-analyses; we assume that in scenarios in which tobacco use prevalence rates decline, 50% of the decline is due to active users becoming former users and the other 50% of the decline is due to never users remaining never users (not initiating use), with linear rates of change from baseline over the course of a decade. The obvious exception to this assumption was brief cessation advice from clinicians, which applied only to active users.

To capture dependence among the risk factors (e.g., to capture the fact that individuals with diabetes are also more likely to have high cholesterol), we use a multivariate normal distribution with the covariance matrix given in SI Table 7.

To update the risk factor profiles between years of the simulation, we carry over pre-existing conditions (diabetes, coronary artery disease, and cerebrovascular disease) from one year to the next and track individuals over time for consistence (e.g., an individual with high blood pressure will continue to have high blood pressure rather than a blood pressure randomly resampled from the population distribution each year), updating their prevalence for age-related and secular trends (SI Table 8). To achieve this consistency between years, we record a variable that captures the rank of each individual’s risk in the cohort (e.g., the person with highest systolic blood pressure has rank #1 in the systolic blood pressure rank list). Then the individual with the highest risk factor value in one year will get the highest value sampled for that risk factor in the next year, and the individual with the second highest risk factor value will get the second highest sample, etc. This technique prevents survival bias during the subsequent mortality calculation described below, as individuals who are high risk are less likely to survive to later years.

## Hazard calculation

An individual’s risk of myocardial infarction death, stroke death or other death is calculated each year as a function of the individual’s risk profile. The individual’s relative hazard *λ*, the hazard of death from disease *j* in relation to the typical hazard in the cohort that year, is defined by:

(4) $\lambda_{j}=e^{\sum_{i} \beta_{i}x_{i}}$

where *β* is the log of the relative risk of each disease contributed by each risk factor *i* (SI Table 9) and *x* is the value of the risk factor for the individual that year.

To determine individual mortality risk from myocardial infarction, stroke, or other causes of death for a particular year, the population-level cohort- and year-specific mortality rate *ρ* for each disease *j* is multiplied by the ratio of the individual’s relative hazard *λ* and the mean relative hazard *ψ* in that individual’s cohort that year for that disease:

(5) $\kappa_{j}=\rho_{j}\frac{\lambda_{j}}{\psi_{j}}$

where *κ* is the mortality rate for the given disease *j* (coronary disease, cerebrovascular disease, or other mortality cause) for the individual that year (SI Tables 10 and 11). The probability of an individual’s death in a given year is given by the sum of the individual’s yearly mortality risk from myocardial infarction, stroke and other causes. A competing risks algorithm proceeds as follows: suppose the death rate for a specific individual (in our model, based on age, sex, and urban/rural location in India) is 0.1/year from cause A and 0.2/year from an aggregate set of all other causes (total all-cause mortality minus mortality attributable to cause A). Thus total all-cause mortality is 0.3/year. A random number is sampled from the uniform distribution between 0 and 1; if less than 0.1, the the individual dies of cause A; if between 0.1 and 0.3, the individual dies of a non-type-A cause; and if greater than 0.3, the individual does not die that year. By sampling from a uniform distribution, the competing risks algorithm avoids biases that would result if we programmed the model to first simulate CVD-related deaths, then other all-caused deaths among survivors of CVD, or vice versa. This also allows all-cause death to change independently of CVD-specific mortality, such that non-CVD deaths are assumed constant, subject to secular trends as we specify in the SI Tables. The simulations were repeated 10,000 times to estimate mean mortality rates and 95% confidence intervals around each mortality rate estimate. All simulations were performed in MATLAB version R2012a (Cambridge, The MathWorks, Inc.). For a validation of this approach, see the SI monograph of the Institute for Health Metrics and Evaluation report ([1](#_ENREF_1)).

# References

1. Lim SS, Gaziano TA, Gakidou E, Reddy KS, Farzadfar F, Lozano R, et al. Prevention of cardiovascular disease in high-risk individuals in low-income and middle-income countries: health effects and costs. Lancet 2007;370(9604):2054-62.

2. Registrar General & Census Commissioner. Census of India. Delhi: Ministry of Home Affairs; 2011.

3. World Health Organization. WHO Global InfoBase. Geneva: WHO; 2012.

4. The MathWorks. Simulating Dependent Random Variables Using Copulas. Cambridge: MathWorks; 2012.

5. World Health Organization. Study on Global AGEing and Adult Health (SAGE). Geneva: WHO; 2011.

6. Tobacco Free Initiative. Global Adult Tobacco Survey (GATS) India Report 2009-2010. Geneva: WHO; 2011.

7. Sadikot SM, Nigam A, Das S, Bajaj S, Zargar AH, Prasannakumar KM, et al. The burden of diabetes and impaired glucose tolerance in India using the WHO 1999 criteria: prevalence of diabetes in India study (PODIS). Diabetes research and clinical practice 2004;66(3):301-7.

8. Danaei G, Finucane MM, Lu Y, Singh GM, Cowan MJ, Paciorek CJ, et al. National, regional, and global trends in fasting plasma glucose and diabetes prevalence since 1980: systematic analysis of health examination surveys and epidemiological studies with 370 country-years and 2.7 million participants. Lancet 2011;378(9785):31-40.

9. Shah B, Kumar N, Menon G, Khurana S, Kumar H. Assessment of burden of non-communicable diseases. Delhi: WHO India; 2010.

10. Teo KK, Ounpuu S, Hawken S, Pandey MR, Valentin V, Hunt D, et al. Tobacco use and risk of myocardial infarction in 52 countries in the INTERHEART study: a case-control study. Lancet 2006;368(9536):647-58.

11. Barnoya J, Glantz SA. Cardiovascular effects of secondhand smoke: nearly as large as smoking. Circulation 2005;111(20):2684-98.

12. World Health Organization. Disease and injury country estimates. Geneva: WHO; 2008.

13. Gaziano TA, Opie LH, Weinstein MC. Cardiovascular disease prevention with a multidrug regimen in the developing world: a cost-effectiveness analysis. Lancet 2006;368(9536):679-86.
